# Reconstruction of Ancestral Metabolic Enzymes Reveals Molecular Mechanisms Underlying Evolutionary Innovation through Gene Duplication

**DOI:** 10.1371/journal.pbio.1001446

**Published:** 2012-12-11

**Authors:** Karin Voordeckers, Chris A. Brown, Kevin Vanneste, Elisa van der Zande, Arnout Voet, Steven Maere, Kevin J. Verstrepen

**Affiliations:** 1 VIB Laboratory for Systems Biology, Leuven, Belgium; 2 CMPG Laboratory for Genetics and Genomics, KU Leuven, Leuven, Belgium; 3 Fathom Information Design, Boston, Massachusetts, United States of America; 4 Faculty of Arts and Sciences Center for Systems Biology, Harvard University, Cambridge, Massachusetts, United States of America; 5 Department of Chemistry and Chemical Biology, Harvard University, Cambridge, Massachusetts, United States of America; 6 VIB Department of Plant Systems Biology, Gent, Belgium; 7 Department of Plant Biotechnology and Bioinformatics, Ghent University, Gent, Belgium; 8 Laboratory for Molecular en Structural Biology, KU Leuven, Leuven, Belgium; University of Chicago, United States of America

## Abstract

Gene duplications are believed to facilitate evolutionary innovation. However, the mechanisms shaping the fate of duplicated genes remain heavily debated because the molecular processes and evolutionary forces involved are difficult to reconstruct. Here, we study a large family of fungal glucosidase genes that underwent several duplication events. We reconstruct all key ancestral enzymes and show that the very first preduplication enzyme was primarily active on maltose-like substrates, with trace activity for isomaltose-like sugars. Structural analysis and activity measurements on resurrected and present-day enzymes suggest that both activities cannot be fully optimized in a single enzyme. However, gene duplications repeatedly spawned daughter genes in which mutations optimized either isomaltase or maltase activity. Interestingly, similar shifts in enzyme activity were reached multiple times via different evolutionary routes. Together, our results provide a detailed picture of the molecular mechanisms that drove divergence of these duplicated enzymes and show that whereas the classic models of dosage, sub-, and neofunctionalization are helpful to conceptualize the implications of gene duplication, the three mechanisms co-occur and intertwine.

## Abstract 2

Resurrection of ancient fungal maltase enzymes uncovers the molecular details of how repeated gene duplications allow the evolution of protein variants with different functions.

## Author Summary

Darwin's theory of evolution is one of gradual change, yet evolution sometimes takes remarkable leaps. Such evolutionary innovations are often linked to gene duplication through one of three basic scenarios: an extra copy can increase protein levels, different ancestral subfunctions can be split over the copies and evolve distinct regulation, or one of the duplicates can develop a novel function. Although there are numerous examples for all these trajectories, the underlying molecular mechanisms remain obscure, mostly because the preduplication genes and proteins no longer exist. Here, we study a family of fungal metabolic enzymes that hydrolyze disaccharides, and that all originated from the same ancestral gene through repeated duplications. By resurrecting the ancient genes and proteins using high-confidence predictions from many fungal genome sequences available, we show that the very first preduplication enzyme was promiscuous, preferring maltose-like substrates but also showing trace activity towards isomaltose-like sugars. After duplication, specific mutations near the active site of one copy optimized the minor activity at the expense of the major ancestral activity, while the other copy further specialized in maltose and lost the minor activity. Together, our results reveal how the three basic trajectories for gene duplicates cannot be separated easily, but instead intertwine into a complex evolutionary path that leads to innovation.

## Introduction

In a seminal book, Susumu Ohno argued that gene duplication plays an important role in evolutionary innovation [1]. He outlined three distinct fates of retained duplicates that were later formalized by others (for reviews, see [2],[3]). First, after a duplication event, one paralog may retain the ancestral function, whereas the other allele may be relieved from purifying selection, allowing it to develop a novel function (later called “neofunctionalization”). Second, different functions or regulatory patterns of an ancestral gene might be split over the different paralogs (later called “subfunctionalization” [4],[5]). Third, duplication may preserve the ancestral function in both duplicates, thereby introducing redundancy and/or increasing activity of the gene (“gene dosage effect” [6]).

Recent studies have shown that duplications occur frequently during evolution, and most experts agree that many evolutionary innovations are linked to duplication [7]–[10]. A well-known example are crystallins, structural proteins that make up 60% of the protein in the lenses of vertebrate eyes. Interestingly, paralogs of many crystallins function as molecular chaperones or glycolytic enzymes. Studies suggest that on multiple occasions, an ancestral gene encoding a (structurally very stable) chaperone or enzyme was duplicated, with one paralog retaining the ancestral function and one being tuned as a lens crystallin that played a crucial role in the optimization of eyesight [11],[12].

The molecular mechanisms and evolutionary forces that lead to the retention of duplicates and the development of novel functions are still heavily debated, and many different models leading to Ohno's three basic outcomes have been proposed (reviewed in [2],[3],[13],[14]). Some more recent models blur the distinction between neo- and subfunctionalization [15]. Co-option models, for example, propose that a novel function does not develop entirely de novo but originates from a pre-existing minor function in the ancestor that is co-opted to a primary role in one of the postduplication paralogs [2],[13]. Examples of such co-option models include the “gene sharing” or “Escape from Adaptive Conflict” (EAC) model [5],[16]–[19] and the related “Innovation, Amplification and Divergence” (IAD) model [20]–[22]. The IAD model describes co-option as a neofunctionalization mechanism. A “novel” function arises in the preduplication gene, and increased requirement for this (minor) activity is first met by gene amplification (e.g., through formation of tandem arrays). After this, adaptive mutations lead to divergence and specialization of some of the duplicate copies. The EAC model, on the other hand, describes co-option rather as a subfunctionalization mechanism by which duplication allows a multifunctional gene to independently optimize conflicting subfunctions in different daughter genes.

Another aspect in which various models differ is the role of positive selection. Some models emphasize the importance of neutral drift, while in other models adaptive mutations play an important role. For example, in the Duplication-Degeneration-Complementation (DDC) model of subfunctionalization [4], degenerative mutations (accumulated by neutral drift) lead to complementary loss-of-function mutations in the duplicates, so that both copies become essential to perform all of the functions that were combined in the single preduplication gene. Whereas this type of subfunctionalization only involves genetic drift [4],[8], other subfunctionalization models, such as the EAC model, attribute an important role to positive selection for the further functional optimization of the postduplication paralogs [2],[14].

There is a sharp contrast between the large number of detailed theoretical models of evolution after gene duplication, on the one hand, and the lack of clear experimental evidence for the various predictions made by these theories, on the other [2]. The key problem is the lack of knowledge about the functional properties of the ancestral, preduplication gene. Since these ancient genes and the proteins they encode no longer exist, many details in the chain of events that led from the ancestral gene to the present-day duplicates remain obscure. In most studies, the activities of the preduplication ancestor are inferred from unduplicated present-day outgroup genes that are assumed to have retained similar functional properties, but this is only an approximation. The central hurdle to surpass to obtain accurate experimental data on the evolution of gene duplicates involves rewinding the evolutionary record to obtain the sequence and activity of the ancestral proteins. Recent developments in sequencing and bio-informatics now enable us to reconstruct ancestral genes and proteins and characterize them in detail [23]–[31]. However, most ancestral reconstruction studies to date did not focus on the mechanisms that govern evolution after gene duplication.

In this study, we used the yeast *MALS* gene family as a model system to gain insight in the molecular mechanisms and evolutionary forces shaping the fate of duplicated genes. The *MALS* genes encode α-glucosidases that allow yeast to metabolize complex carbohydrates like maltose, isomaltose, and other α-glucosides [32],[33]. Several key features make this family ideal to study duplicate gene evolution. First, it is a large gene family encompassing multiple gene duplication events, some ancient and some more recent. Second, the present-day enzymes have diversified substrate specificities that can easily be measured [32]. Third, the availability of *MALS* gene sequences from many fungal genomes enabled us to make high-confidence predictions of ancestral gene sequences, resurrect key ancestral proteins, and study the selective forces acting throughout the evolution of the different gene duplicates. Fourth, the crystal structure of one of the present-day enzymes, Ima1, has been determined [34]. Molecular modeling of the enzymes' binding pocket, combined with activity measurements on reconstructed and present-day enzymes, allowed us to investigate how mutations altered enzyme specificity and gave rise to the present-day alleles that allow growth on a broad variety of substrates. Combining these analyses, we were able to study the evolution and divergence of a multigene family to an unprecedented level of detail and show that the evolutionary history of the *MALS* family exhibits aspects of all three classical models of duplicate gene evolution proposed by Ohno (gene dosage, neo-, and subfunctionalization).

## Results

### The Present-Day Maltase Enzymes Arose from a Functionally Promiscuous Ancestor

**Figure 1:**
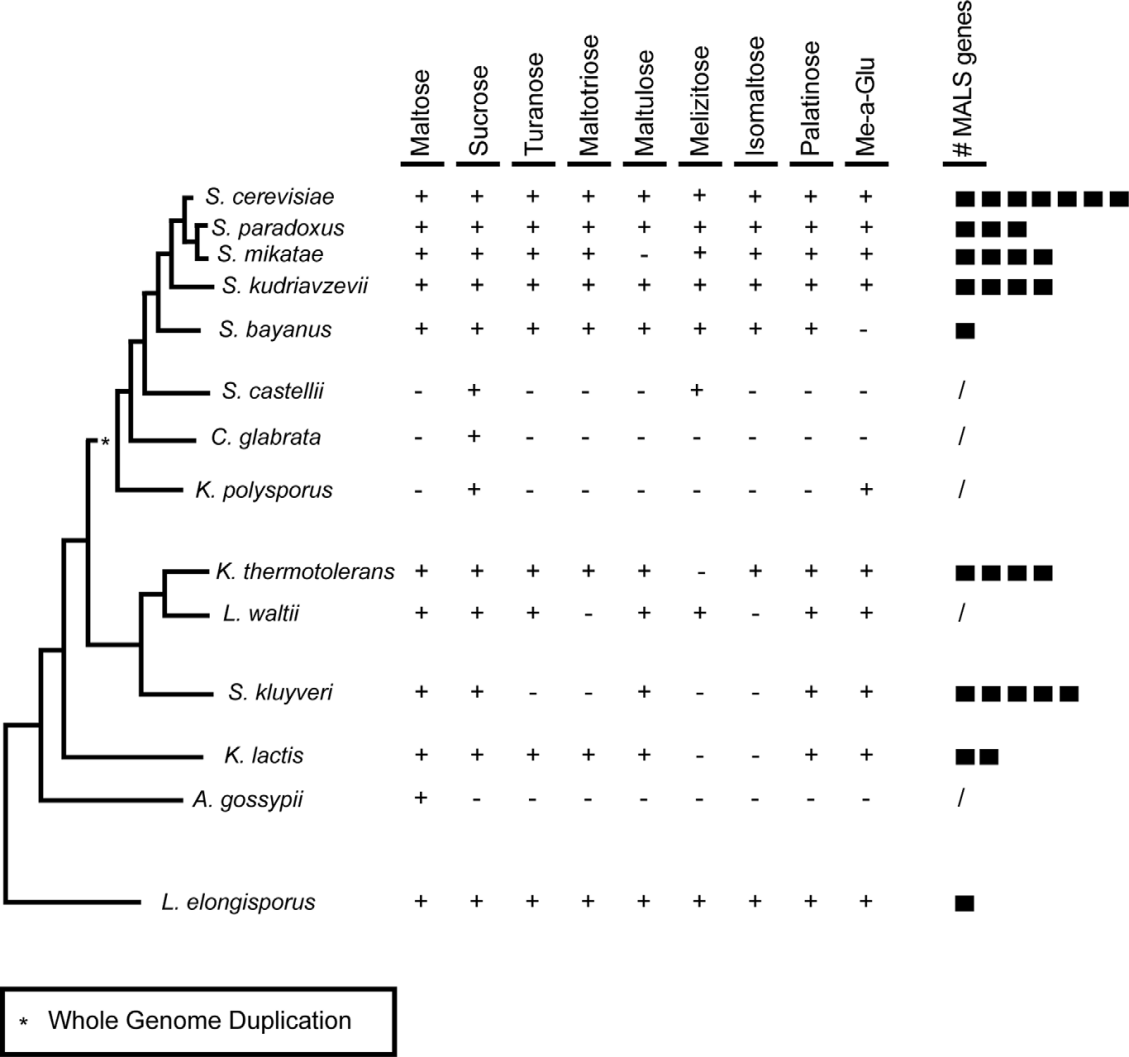
Yeast species can grow on a broad spectrum of α-glucosides. Serial dilutions of each species were spotted on medium (Yeast Nitrogen Base w/o amino acids) with 2% of each sugar (Me-a-Glu  =  methyl-α-glucoside). Growth was scored after 3 d incubation at 22°C. +, growth; −, no growth. # *MALS* genes, the number of maltase genes found in each of these strains. Genotypes are listed in Table S5.

Some yeast species have evolved the capacity to metabolize a broad spectrum of natural disaccharides found in plants and fruits (Figure 1, tree adapted from [35]). The origin of this evolutionary innovation seems to lie in the duplication and functional diversification of genes encoding permeases and hydrolases [32]. The common *Saccharomyces cerevisiae* laboratory strain S288c, for example, contains seven different *MALS* genes (*MAL12*, *MAL32*, and *IMA1–5*), which originated from the same ancestral gene but allow growth on different substrates [32],[33].

To understand how duplications led to functionally different MalS enzymes, we reconstructed, synthesized, and measured the activity of key ancestral MalS proteins. We used the amino acid (AA) sequences of 50 maltases from completely sequenced yeast species, ranging from *Saccharomyces cerevisiae* to *Pichia* and *Candida* species, for phylogenetic analysis and ancestral sequence reconstruction (see Materials and Methods and Dataset S1). A consensus amino-acid-based phylogenetic tree was constructed using MrBayes [36] under the LG+I+G model with four rate categories (see Figure S1, and see Materials and Methods for details). Trees constructed using MrBayes under other models of sequence evolution (WAG, JTT) generated largely identical results (unpublished data). To further check the robustness of the AA tree inferred by MrBayes, we inferred a maximum likelihood (ML) tree under the LG+I+G model using PhyML (Figure S2) [37]. With the exception of a few recent splits in the topology, the MrBayes and PhyML trees agree, increasing our confidence in the constructed tree. Codon-based tree reconstruction using MrBayes yielded similar results (see further). Additional tests were performed to control for potential long branch attraction (LBA) artifacts, specifically to check the placement of the *K. lactis* branch as an outgroup to the *Saccharomyces* and *Lachancea* clades (see Text S1 and Figures S3, S4, S5, S6).

Next, we reconstructed the AA sequence of the ancestral maltases under several commonly used models of protein evolution (LG, WAG, JTT; see Materials and Methods). All models support roughly the same ancestral protein sequences, increasing our confidence in the reconstructed ancestral sequences. In particular, all models identified the same residues for variable sites within 10 Å of the active center (based on the crystal structure of the Ima1 protein), which are likely relevant sites with respect to enzymatic activity. The residues for a few other sites located further away from the active pocket vary between different models, but differences generally involve biochemically similar AAs (see Table S1).

Synthesis of the ancestral enzymes was based on the reconstructed ancestral sequences obtained with the JTT model. For ambiguous residues (i.e., sites for which the probability of the second-most likely AA is >0.2) within 7.5 Å of the binding pocket, we constructed proteins containing each possible AA, while for ambiguous residues outside 7.5 Å we considered only the most likely AA. There is one ambiguous residue close to the active center in the ancestral proteins ancMalS and ancMal-Ima, namely residue 279 (based on *Saccharomyces cerevisiae* S288c Ima1 numbering). We therefore synthesized two alternative versions of these proteins, one having G and one having A at position 279. Whereas these alternative proteins show different activities for some substrates, the relative activities are similar and our conclusions are robust. Sequences for these reconstructed enzymes can be found in Dataset S2. In the main figures, we show the variant with the highest confidence. Enzymatic data for all variants can be found in Table S2.

**Figure 2:**
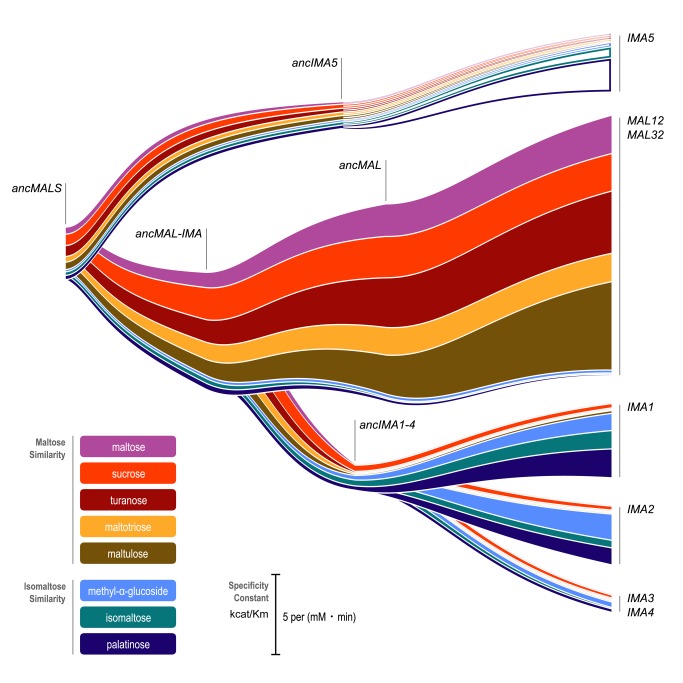
Duplication events and changes in specificity and activity in evolution of *S. cerevisiae* MalS enzymes. The hydrolytic activity of all seven present-day alleles of Mal and Ima enzymes as well as key ancestral (anc) versions of these enzymes was measured for different α-glucosides. The width of the colored bands corresponds to k_cat_/K_m_ of the enzyme for a specific substrate. Specific values can be found in Table S2. Note that in the case of present-day Ima5, we were not able to obtain active purified protein. Here, the width of the colored (open) bands represents relative enzyme activity in crude extracts derived from a yeast strain overexpressing *IMA5* compared to an *ima5* deletion mutant. While these values are a proxy for the relative activity of Ima5 towards each substrate, they can therefore not be directly compared to the other parts of the figure. For ancMalS and ancMal-Ima, activity is shown for the variant with the highest confidence (279G for ancMalS and 279A for ancMal-Ima). Activity for all variants can be found in Table S2.

The activity of all resurrected ancestral enzymes was determined for different substrates (see Materials and Methods, Text S1, and Figure 2). The results indicate that the very first ancestral enzyme, denoted as ancMalS, was functionally promiscuous, being primarily active on maltose-like substrates but also having trace activity on isomaltose-like sugars. The activity data presented in Figure 2 show how this promiscuous ancestral protein with relatively poor activity for several substrates evolved to the seven present-day enzymes that show high activity for a subset of substrates, and little or no activity for others. This confirms the existence of two functional classes of MalS enzymes that originated from ancient duplication events. First, Mal12 and Mal32 show activity against maltose-like disaccharides often encountered in plant exudates, fruits, and cereals, like maltose, maltotriose, maltulose, sucrose, and turanose (a signaling molecule in plants). The five MalS enzymes of the second class (Ima1–5), which in fact result from two independent ancient duplication events giving rise to the Ima1–4 and Ima5 clades, show activity against isomaltose-like sugars including palatinose (found in honey [38]) and isomaltose. Differences in hydrolytic activity between members of the same (sub)class are more subtle or even absent, which is not surprising since some of these recent paralogs are nearly identical (Mal12 and Mal32, for example, are 99.7% identical on the AA level).

The more recent ancestral enzymes also show a similar split in activity, with some enzymes (ancMal) showing activity towards maltose-like substrates, and others (ancIma1–4) towards isomaltose-like substrates. Moreover, activity on isomaltose-like sugars (isomaltose, palatinose, and methyl-α-glucoside) changes in a coordinate fashion when comparing different enzymes, and the maltose-like sugars also group together. Careful statistical analysis reveals that the maltose-like group consists of two subgroups (maltose, maltotriose, maltulose, and turanose, on one hand, and sucrose, on the other) that behave slightly different, showing that the enzymes show quantitative differences in the variation of specificity towards these substrates (two-way ANOVA analysis followed by Games-Howell test on log-transformed k_cat_/K_m_ values; *p* values can be found in Table S3).

Interestingly, the most ancient ancestral enzymes do not show a clear split in activity towards either maltose-like or isomaltose-like sugars after duplication, and the transition of ancMalS to ancMal-Ima even shows an increase in activity for all substrates. This suggests that (slight) optimization for all substrate classes simultaneously was still possible starting from ancMalS. A clear divergence of both subfunctions occurred later, after duplication of ancMal-Ima, resulting in ancMal and ancIma1–4. AncMal shows a significant increase in activity on maltose-like sugars accompanied by a significant drop in activity on isomaltose-like sugars compared to ancMal-Ima; and the reverse is true for ancIma1–4 (see also Table S3 for exact *p* values for each enzyme–enzyme comparison on the different sugars tested). Together, this illustrates how, after duplication, the different copies diverged and specialized in one of the functions present in the preduplication enzyme.

In two separate instances, a major shift in specificity is observed, from maltose-like sugars to isomaltose-like sugars (transition from anc*IMA5* to *IMA5*, and from anc*MAL-IMA* to anc*IMA1–4*). The shift in activity from anc*MAL-IMA* to anc*IMA1–4* is particularly pronounced. The anc*MAL-IMA* enzyme hydrolyzes maltose, sucrose, turanose, maltotriose, and maltulose but has hardly any measurable activity for isomaltose and palatinose, whereas anc*IMA1–4* can only hydrolyze isomaltose and palatinose (and also sucrose). For the evolution of the maltase-like activity from the ancestral MalS enzyme to the present-day enzyme Mal12, we see a 2-fold increase in k_cat_ and a 3-fold decrease in K_m_ for maltose, indicating an increase in both catalytic power and substrate affinity for this sugar. For the evolution of isomaltase-like activity in the route leading to Mal12, k_cat_ decreases more than 3-fold for methyl-α -glucoside. k_cat_ for isomaltose and palatinose and the affinity for isomaltose and palatinose are so low that they could not be measured (see Table S2 for the exact values of k_cat_ and K_m_ for each enzyme and each sugar; results of two-way ANOVA analysis followed by Games-Howell test comparing log-transformed k_cat_/K_m_ values for different enzymes on each of the sugars can be found in Table S3).

### Present-Day Enzymes from Other Yeast Species Show Similar Patterns of Functional Diversification

**Figure 3:**
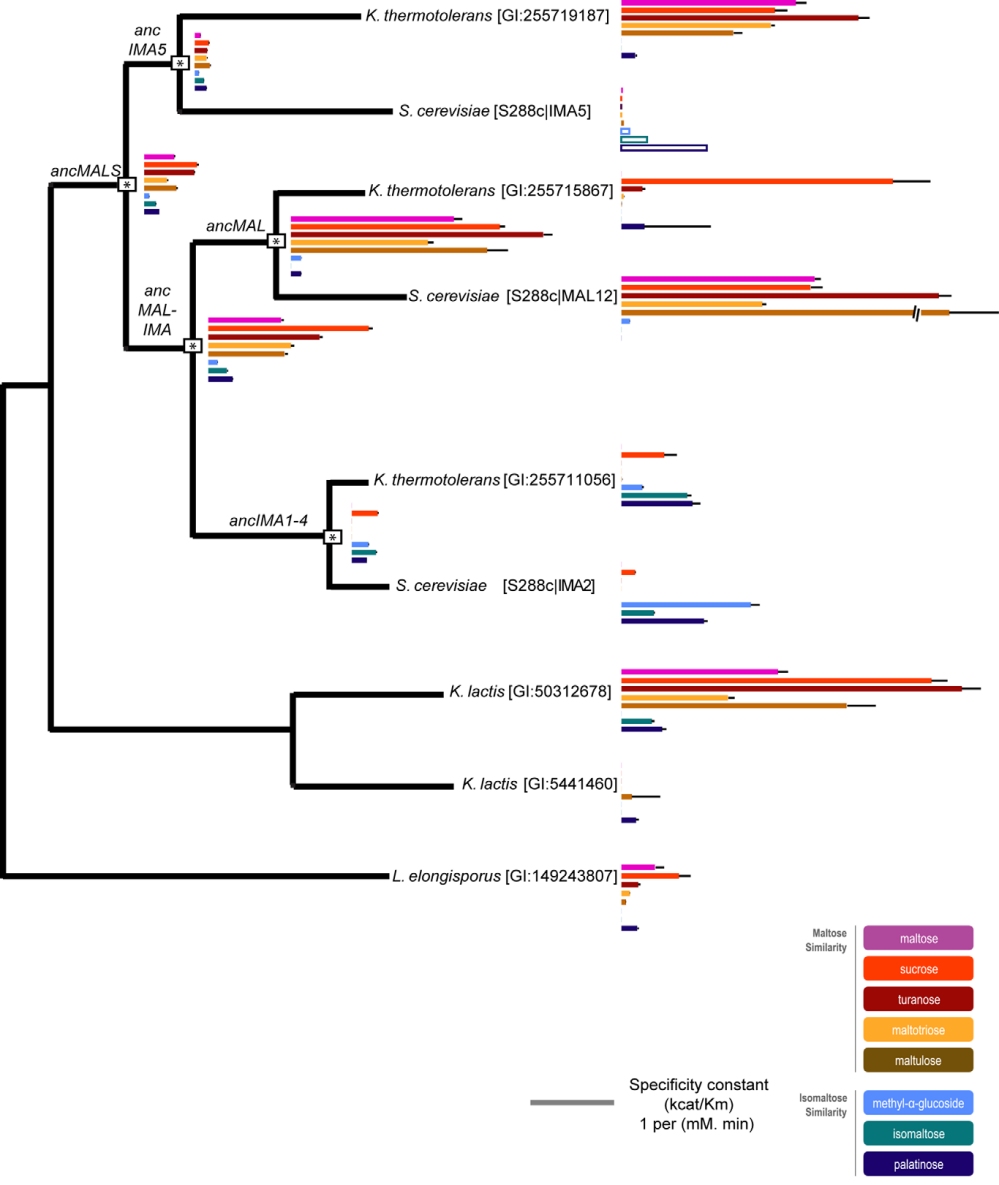
Activities of present-day Mal enzymes in distant fungi correspond well with activities of reconstructed ancestral enzymes. Basic phylogeny of the *MALS* gene family with different clades, showing the ancestral bifurcation points (indicated by *). Length of the colored bands corresponds to the measured k_cat_/K_m_ of the enzyme for a specific substrate. Bands for Ima5 represent relative enzyme activity in crude extracts derived from a yeast strain overexpressing *IMA5* compared to an *ima5* deletion mutant. For ancMalS and ancMal-Ima, activity is shown for the variant with the highest confidence (279G for ancMalS and 279A for ancMal-Ima). Error bars represent standard deviations. Activity for all variants and the corresponding standard deviations can be found in Table S2.

**Figure 4:**
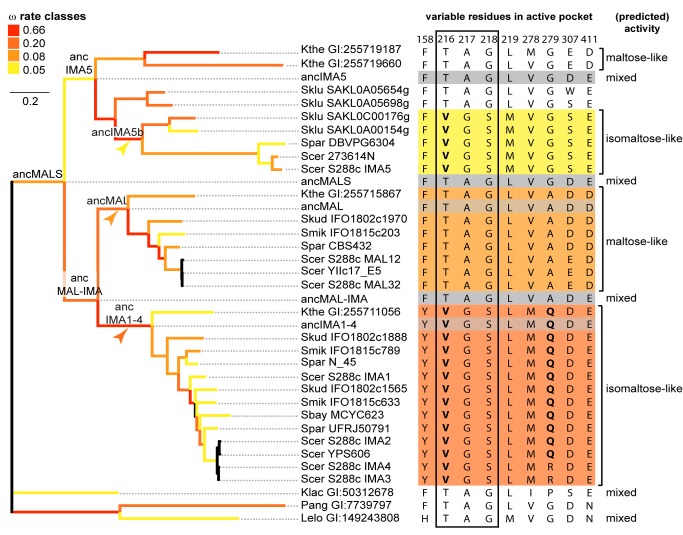
Positive selection on residues near binding pocket resulted in distinct subgroups with different substrate preference. An unrooted codon-based phylogenetic tree of the *MALS* gene family is shown on the left. Branches are colored according to the ω (dN/dS) rate classes inferred from GA Branch analysis [41]. Branches for which branch-site tests for positive selection were performed are indicated by colored arrowheads. Since ω rate classes cannot be inferred reliably for very small branches, branches <0.01 are not colored. The right part of the figure shows the nine variable AA residues located near the substrate binding pocket of the respective enzymes (numbering based on Ima1 sequence). Sequences of ancestral enzymes are shaded in grey. Subgroups of enzymes that show similar substrate specificity are colored accordingly. Residues indicated in bold were found to be under positive selection by the branch-site tests. Perfectly co-varying residues are boxed. Substrate preference of extant and ancestral enzymes was deduced from enzyme assays on *S. cerevisiae*, *K. lactis*, *K. thermotolerans*, *L. elongisporus*, and reconstructed ancestral enzymes (see Figure 3 and Table S4).

To further explore the evolution of *MALS* genes and consolidate the measured activities of the ancestral enzymes, we expressed and purified additional present-day α-glucosidase alleles from other yeast species and measured their activities (Figure 3). We focused primarily on enzymes that are directly related to one of the ancestral proteins but did not undergo any further duplication events, and therefore have a higher probability of having retained a similar activity as their (sub)class ancestor. Indeed, the only present-day MalS enzyme of the yeast *L. elongisporus* has a broad but relatively weak activity comparable to the very first ancestral MalS enzyme, providing extra support for the accuracy of our ancestral reconstructions. Also in *K. lactis*, which contains two Mal alleles, one of the paralogs retains the broad specificity of ancMalS. The other paralog (GI:5441460) has a deletion of five AAs close to the active pocket that likely explains the general lack of activity of this enzyme (see Materials and Methods and Figure S7). In contrast, yeasts that show multiple duplication events, like *K. thermotolerans* and *S. cerevisiae*, exhibit specialization, with some enzymes showing only activity for maltose-like substrates and others for isomaltose-like substrates. Moreover, the activities (maltase- or isomaltase-like) of homologs in *S. cerevisiae* and *K. thermotolerans* derived from the same intermediate ancestor are often similar, except in the *IMA5* clade. Here, the *K. thermotolerans* and *S. cerevisiae* homologs have very different substrate specificities, indicating species-specific evolutionary trajectories and/or reciprocal paralog loss in the different species (Figures 3 and 4).

### Molecular Modeling and Resurrection of Ancestral Proteins Identify Residue 279 in the Enzymes' Binding Pocket as a Key Determinant of Substrate Specificity

Next, we investigated which mutations underlie the observed functional changes. We used the recently resolved crystal structure of Ima1 (pdb entry 3A4A) [34] as a template to study the molecular structure of the enzymes' substrate binding pocket (see Materials and Methods). All enzymes share a highly conserved molecular fold, suggesting that changes in activity or substrate preference are likely caused by mutations in or around the substrate binding pocket. We identified nine variable AA residues within 10 Å of the center of the binding pocket in the various paralogs (Figure 4, right panel). Site-directed mutagenesis and crystallographic studies by Yamamoto et al. confirmed the importance of several of these residues for substrate specificity in the present-day Ima1 protein [39],[40]. In particular, Yamamoto et al. [40] characterized the influence of residues 216-217-218 (Ima1 numbering), which covary perfectly with each other and with the observed substrate specificity shifts across the phylogeny presented in Figure 4. Sequence co-evolution analysis on 640 *MAL12* homologs identified another cluster of three co-evolving residues among these nine residues (positions 218, 278, and 279 in Ima1), which we investigate here in detail.

**Figure 5:**
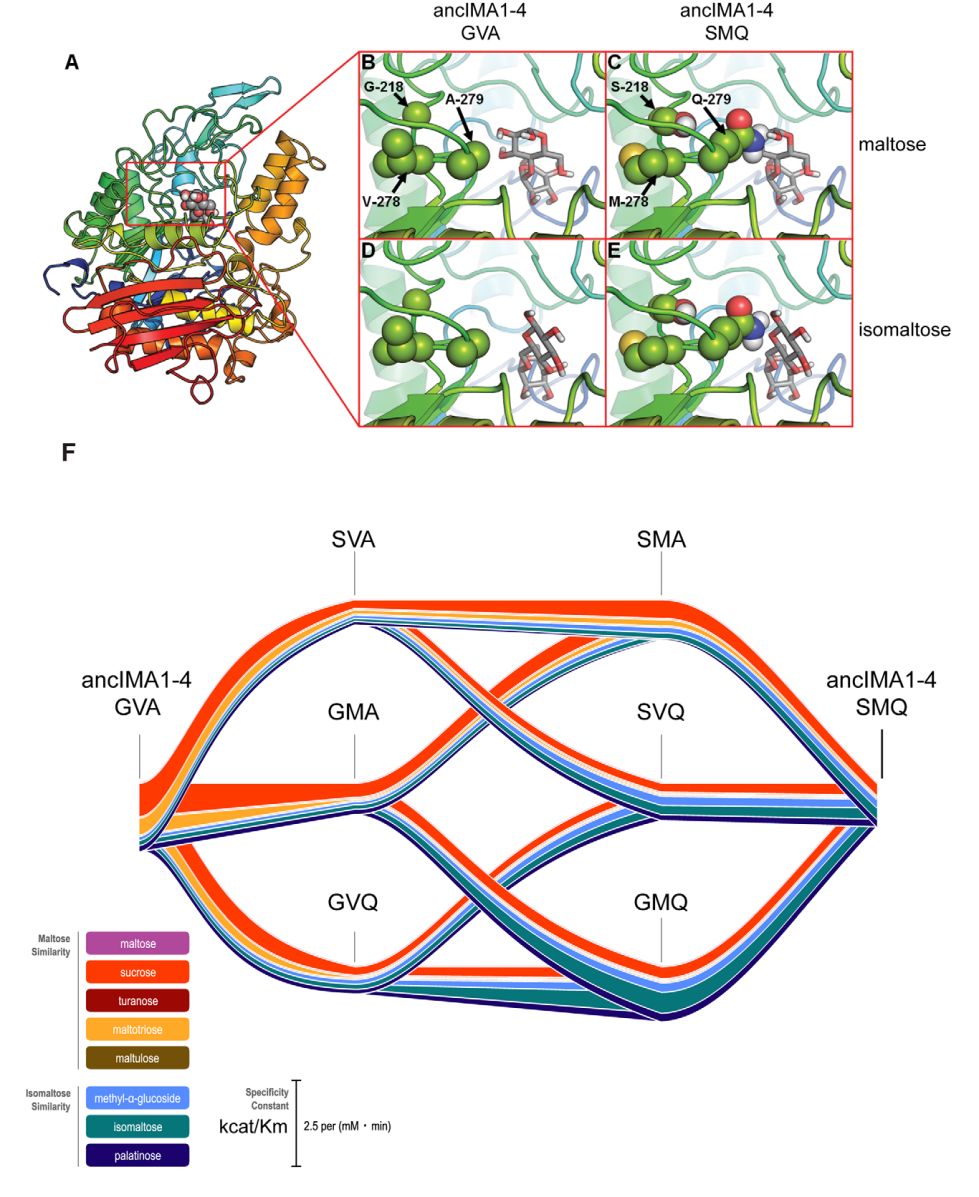
Three co-evolving residues determine the shift in activity observed in the evolution of Ima1–4. (A) Global structure of the MalS proteins with maltose, represented as spheres, bound in the active site. Panels (B–E) show details of the active site, with substrates as sticks (maltose in panels B and C; isomaltose in panels D and E). The variable AAs are shown as spheres. Structural analysis of the binding site suggests that the A279Q mutation affects substrate specificity the most. The side chain of Q279 sterically hinders binding of maltose but stabilizes isomaltose binding through polar interactions. The G218S and V278M changes cause subtle adaptations of the fold, causing Q279 to protrude further into the binding pocket, which allows optimal interaction with isomaltose. (F) Activity (k_cat_/K_m_) of all possible intermediary forms in the evolution of three co-evolving residues in AncIma1–4, obtained from enzyme assays performed for all reconstructed proteins. Values for k_cat_ and K_m_ can be found in Table S2.

**Figure 6:**
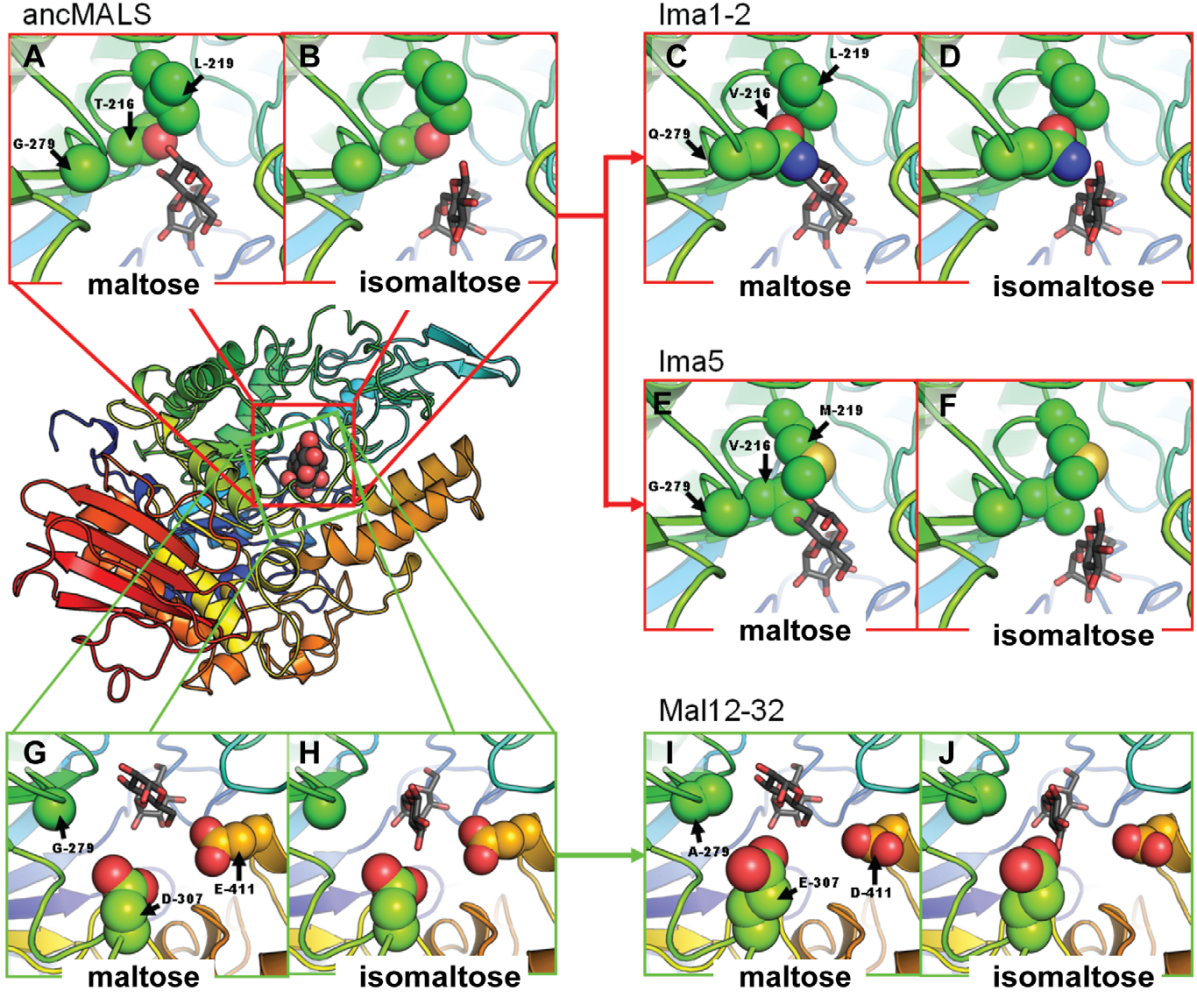
Evolution of the promiscuous AncMalS enzyme into isomaltose- and maltose-hydrolyzing enzymes. AncMalS is a promiscuous enzyme that hydrolyzes both maltose- and isomaltose-like substrates, whereas the present-day enzymes Ima1,2 and Ima5 preferentially hydrolyze isomaltose-like sugars and Mal12–32 preferentially hydrolyzes maltose-like sugars. First, the presence of a Thr or Val residue at position 216 affects the binding affinity of the enzyme through changes in the hydrophobic/hydrophilic interactions with the different substrate classes (panels A to D; see also Figure S8). The case of Ima1,2 and Ima5 (panels C to F) illustrates that an additional shift in substrate specificity can be obtained via different evolutionary routes. In the case of Ima1 and Ima2, the change of G279 to Q279 interferes with binding of maltose-like substrates, but the side chain of Gln can undergo polar interactions with isomaltose (panels C and D). The G218S and V278M changes cause additional subtle adaptations of the protein fold, causing Q279 to protrude further into the binding pocket, allowing optimal interaction with isomaltose (see also Figure 5). The evolution of isomaltase activity in Ima5 also occurred via the introduction of steric hindrance in the binding pocket, although in this case the change involved was L219M (panels E and F). In ancMalS, residues D307 and E411 allow binding of both maltose- and isomaltose-like substrates (panels G and H). In the maltose-specific enzymes Mal12 and Mal32, however, these residues have evolved to E307 and D411 (panels I and J). These changes not only increase the affinity for maltose-like substrates but also make this site incompatible with isomaltose-like substrates. Subpanels are graphical representations of the binding pocket, with key amino acids depicted as spheres. Maltose and isomaltose are represented as sticks.

Together with residues 216 and 217, residues 218, 278, and 279 seem to contribute to the activity shift observed in the evolution of Ima1–4 (see Figures 4–6, Figure S8, and Supplementary Information for details). Molecular modeling of the mutations at 218-278-279 on the branch leading to ancIma1–4 (see Figure 4) suggests that the change from alanine to glutamine at residue 279 shifts the binding preference of the pocket from maltose-like to isomaltose-like sugars (Figure 5B–E). The two co-evolving residues at positions 218 and 278 are spatially close to AA 279 and cause subtle structural adaptations that help to better position the Q residue.

To investigate if changes at all three positions are necessary for the observed shift in substrate specificity from anc*MAL-IMA* to anc*IMA1–4* and to investigate the possible evolutionary paths leading to these three interdependent mutations, we synthesized all possible intermediate anc*IMA1–4* enzyme variants with mutations at positions 218, 278, and 279. We subsequently expressed, purified, and measured activity of these enzyme variants. Figure 5F depicts the results of these enzyme assays and shows that these residues indeed affect substrate specificity, with the largest shift depending on the A to Q change at position 279, as expected from structural analysis. For one mutational path (GVA to GVQ to SVQ to SMQ), we observe a gradual increase in activity towards isomaltose and palatinose, demonstrating that there is a mutational path that leads to a consistent increase in isomaltase activity without traversing fitness valleys. Moreover, in keep with the stabilizing role of the mutations at positions 218 and 278, the A to Q change at position 279 along this path takes place before the two other mutations at positions 218 and 278 (Figure 5F).

Besides allowing the development of isomaltase activity in the Ima proteins, duplication also permitted further increase of the major ancestral function (hydrolysis of maltose-like sugars) in Mal12 and Mal32. Structural analysis reveals that this increase in maltase activity, from ancMalS to Mal12/32, is due to mutations D307E and E411D (Figure 6G–J). These mutations increase the fit for maltose-like substrates but also completely block the binding of isomaltose-like substrates (Figure 6). Similar to what is seen for the evolution of AncMal-Ima to AncIma1–4, changes that increase the binding stability of one type of substrate cause steric hindrance that prevents binding of the other class of substrates. These signs of incompatibilities between substrates indicate that it is difficult to fully optimize one enzyme for both maltose-like and isomaltose-like substrates, with the highly suboptimal ancMalS being a notable exception. After partial optimization of ancMalS, duplication of anc*MAL-IMA* likely enabled further optimization of the conflicting activities in separate copies.

### Different Evolutionary Routes Can Lead to Similar Changes in Substrate Specificity

Interestingly, the transition from AncMalS to Ima5 shows a similar shift in substrate specificity as the transition of AncMal-Ima to AncIma1–4. However, the residue at position 279, a key factor in the evolution of AncMal-Ima to AncIma1–4, remains unaltered in the evolution of AncMalS to Ima5. Instead, L219, a residue located proximal to position 279, has changed into M219 in the Ima5 enzyme (Figure 6C–F). How can such seemingly very different mutations yield a similar change in substrate specificity?

Structural analysis shows that the L-to-M mutation at position 219 in Ima5 causes a very similar structural change as the G279Q change in AncIma1–4 (Figure 6), indicating that different evolutionary routes may produce a similar shift in activity. In both cases, the evolution of isomaltase-like activity involved introducing a residue that can stabilize isomaltose-like substrates but causes steric hindrance for maltose-like sugars in the binding pocket. Based on the phylogeny of binding pocket configurations and on our enzyme activity tests, this functional shift in the *IMA5* clade most likely occurred after a duplication in the common ancestor of *S. kluyveri* and *S. cerevisiae* (Figures 3–4).

### Key Residues in Binding Pocket of MalS Enzymes Show Signs of Positive Selection

Next, we investigated the role of selective pressure during the different evolutionary transitions. We used MrBayes to construct a codon-based phylogeny under a GTR codon model of evolution, including 32 *MALS* genes that share the same nuclear genetic code. The resulting codon-based phylogeny was the same as the AA-based phylogeny generated using the LG+I+G protein model for all 50 sequences, apart from two exceptions in the anc*IMA*1–4 clade. First, *S. mikitae IFO1815 c789* and *S. paradoxus N45* branch off separately from *S. kudriavzevii IFO1802 c1888* instead of together. Second, *S. kudriavzevii IFO1802 c1565* now branches off separately instead of multifurcating with *S. mikitae IFO1815 c633* and the branch leading to the *S. cerevisiae IMA2–4* genes. Relative branch lengths between genes were similar to the branch lengths calculated under protein models of evolution. The topology of the codon-based tree is presented in Figure 4.

GA Branch analysis [41] identified a branch class with an elevated ω (dN/dS) rate (ω = 0.66) but did not detect branch classes with ω>1 that would be considered strong proof for positive selection (see Materials and Methods and Figure 4). These results, combined with our activity test results and the observed sequence configurations around the active center, suggest, however, that positive selection might have been operating on specific sites in three specific postduplication branches associated with enzyme activity shifts, namely the anc*IMA*1–4, anc*IMA*5b, and anc*MAL* branches, indicated with arrows on Figure 4. We used the modified branch-site model A implemented in PAML to assess positive selection along these branches (see Materials and Methods) [42]. Results are presented in Table S4. For both the anc*IMA*1–4 and anc*IMA*5b branches, *p* values and parameter estimates suggest that a proportion of sites has strongly elevated ω values, consistent with the GABranch results. On the branch from anc*MAL*-*IMA* to anc*IMA1*–*4*, four sites show signs of positive selection, with a posterior Bayes Empirical Bayes (BEB) probability >0.95, of which two, 216 and 279, are within 10 Å of the active center and known to be important for substrate specificity. On the anc*IMA5*b branch, four sites show signs of positive selection (BEB>0.95), including again site 216. For anc*MAL*, the null model (no positive selection) was not rejected at the 95% significance level. Both the corresponding parameter estimates and results of the GABranch analysis, however, suggest relaxation of purifying constraints on this branch.

To get more support for the PAML branch-site test results, we performed an additional analysis using an alternative branch-site method that was recently implemented in the HyPhy package [43]. This method identified in total seven branches that possibly experienced positive selection: anc*IMA*1–4 (p<0.0001), anc*IMA*5b (*p* = 0.0232), anc*MALS* (*p* = 0.0228), *S. kluyveri SAKL0A05698g* (*p*<0.0001), *K. thermotolerans GI:* 255719187 (*p*<0.0001), the branch leading from anc*IMA*5 to the anc*IMA*5b branch (*p* = 0.0168), and finally the branch leading up to *S. cerevisiae IMA2*, *IMA3*, *IMA4*, and *YPS606* within the anc*IMA*1–4 clade (*p* = 0.0353). In other words, the anc*MALS*, anc*IMA*1–4, and anc*IMA*5b branches are suggested to have evolved under positive selection, together with four other branches. The branch-site method implemented in HyPhy currently does not allow the identification of specific sites that may have evolved under positive selection on these branches.

Together, our analyses indicate that some residues near the active pocket, in particular the key residues 216 and 279 that determine substrate specificity (see above), may have experienced positive selection in the postduplication lineages leading to isomaltose-specific enzymes. It should be noted, however, that the specificity and sensitivity of the currently available methods for detecting positive selection, in particular branch-site methods, is heavily debated [42],[44]–[47]. Possible pitfalls include fallacies in the assumption that synonymous substitutions are neutral, a reported increase in the number of false positives due to sampling errors when the number of (non)synonymous substitutions and sequences is low, and potential inadequacies in the null and alternative models that are being compared, leading to difficulties with completely ruling out other explanations for perceived positive selection. For these reasons, the positive selection test results reported here should be approached as indications rather than definitive proof.

### Recent Duplicates *MAL12* and *MAL32* Are Maintained Because of Gene Dosage Effects

The previous results show how duplication of a promiscuous ancestral enzyme with limited activity towards two substrate categories allowed the evolution of separate enzyme clades that each show increased activities for a specific subset of substrates. The functional diversification of the different clades ensures their retention. However, why are recent, near-identical duplicates such as *MAL12* and *MAL32* conserved?

To investigate if selective pressure might protect the *MAL12*/*MAL32* duplicates, we determined the fitness effect of inactivating each of them. The results in Figure S9 show that strains lacking just one of the *MAL12* and *MAL32* paralogs show a considerable fitness defect compared to a wild-type strain when grown on maltose. These results suggest that gene dosage may play a primary role in preserving these recent paralogs [6]. Dosage effects increasing maltase and/or isomaltase activity may also have played a role after the earliest *MALS* duplications, before the duplicates were optimized for different activities.

## Discussion

One of the major issues in the field of molecular evolution is the plethora of theoretical models and variants of models concerning the evolution of gene duplicates, with few of the claims supported by solid experimental evidence. On many occasions, inherent properties of the evolutionary process make it extremely hard to find or generate experimental evidence for a given model. However, recent developments in genome sequencing, evolutionary genomics, and DNA synthesis open up exciting possibilities. Using these new opportunities, we were able to resurrect ancient *MALS* genes and the corresponding enzymes and provide a detailed picture of the evolutionary forces and molecular changes that underlie the evolution of this fungal gene family. The *MALS* gene family is an ideal model for the study of duplicate gene evolution, since it underwent several duplication events and encodes proteins for which we could accurately measure different activities. The availability of multiple fungal genome sequences provided sufficient data to robustly reconstruct ancestral alleles and study the selective forces that propelled divergent evolution of the paralogs. Additionally, the existence of a high-quality crystal structure of one of the present-day enzymes made it possible to predict the functional effects of mutations and to study the mechanistic basis of suspected adaptive conflicts between the maltase-like and isomaltase-like subfunctions.

**Figure 7:**
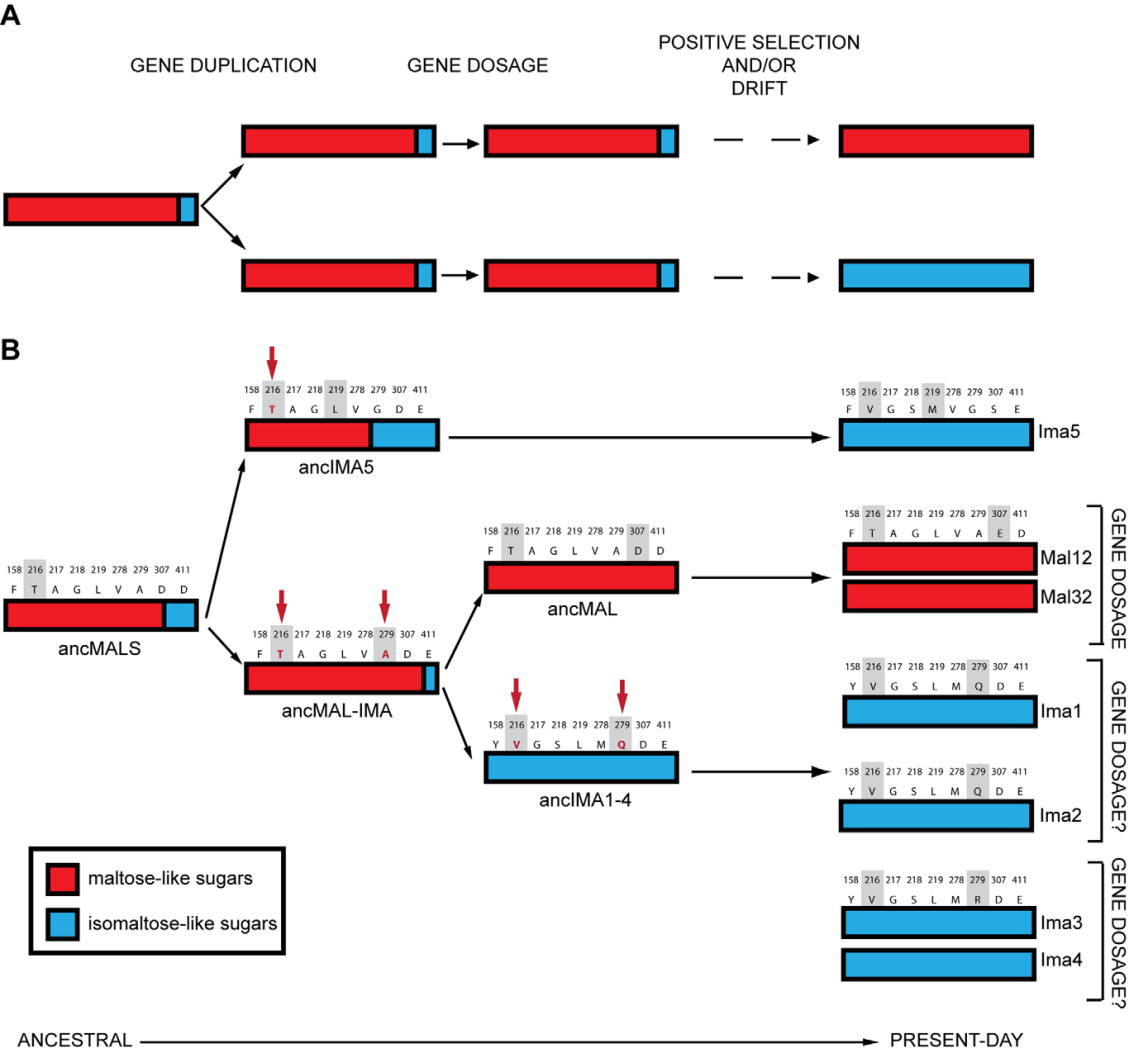
Multiple evolutionary mechanisms contribute to the evolution of the MalS gene family in *S. cerevisiae*. (A) Overview of evolutionary mechanisms in the evolution of an ancestral gene with two conflicting activities (major function, red; minor function, blue). Duplication can help resolve this “adaptive conflict” by allowing optimization of these activities in two separate copies. Increased requirement for either of these activities, for example by changes in the environment, can first be met by duplication of the ancestral gene. Selection for increased gene dosage can help to preserve both copies until adaptive mutations optimize the different functions in separate copies. (B) Evolution of the promiscuous ancestral MalS enzyme into the seven present-day MalS alleles shows how different evolutionary forces contribute to the evolution of gene duplicates. Activity towards isomaltose-like sugars first existed only as a trace activity in the ancestral, preduplication enzyme. The nature of the binding pocket prevented simultaneous optimization of the major and minor function in the ancestral enzyme. Duplication allowed the (full) optimization of the two conflicting activities of the ancestral enzyme in separate copies. Several key residues in the enzymes' binding pocket responsible for these shifts in substrate specificity (shaded in grey) show signs of positive selection (indicated both in red and with red arrows; see also Figure 4). Preservation of more recent, highly similar duplicates like Mal12–Mal32 may be mediated through gene dosage effects (see also Figure S9). Sequences above each enzyme represent the nine variable residues in the binding pocket (numbering based on Ima1 sequence). AA changes that led to improvement of one of the hydrolyzing activities are shaded in grey.

Our results paint a complex and dynamic picture of duplicate gene evolution that combines aspects of dosage selection and sub- and neofunctionalization (see Figure 7). The preduplication ancMalS enzyme was multifunctional and already contained the different activities found in the postduplication enzymes (the basic idea of subfunctionalization), albeit at a lower level. However, the isomaltase-like activity was very weak in the preduplication ancestor and only fully developed through mutations after duplication (increase of k_cat_/K_m_ with one order of magnitude for isomaltase-like substrates from ancMalS to Ima1), which resembles neofunctionalization. The ancestral maltase-like activity also improved substantially but to a lesser extent (factor 6.9 on average from ancMalS to Mal12), which therefore perhaps fits better with the subfunctionalization model. Moreover, our activity tests on Mal12/Mal32 mutants indicate that gene dosage may also have played a role in preserving *MALS* paralogs, especially right after duplication. This may not only have been the case for the recent *MAL12*–*32* and *IMA3*–*4* duplications but also for more ancient duplications involving multifunctional ancestors. In summary, whereas the classical models of dosage, sub-, and neofunctionalization are helpful to conceptualize the implications of gene duplication, our data indicate that the distinction between sub- and neofunctionalization is blurry at best and that aspects of all three mechanisms may intertwine in the evolution of a multigene family.

Although it is difficult to classify our results decisively under one of the many models of evolution after gene duplication, most of our findings agree with the predictions of the “Escape from Adaptive Conflict” (EAC) model [5],[16],[17],[19], a co-option-type model in which duplication enables an organism to circumvent adaptive constraints on a multifunctional gene by optimizing the subfunctions separately in different paralogs. The EAC model makes three key predictions: (i) the ancestral protein was multifunctional, (ii) the different subfunctions could not be optimized simultaneously in the ancestral protein (or at least not in an evolutionarily easily accessible way), and (iii) after duplication, adaptive changes led to optimization of the different subfunctions in separate paralogs [13],[16],[48]. In general, our findings fit with these predictions: (i) we find that several of the ancestral preduplication maltase enzymes (anc*MALS*, anc*MAL-IMA*, and anc*IMA5*) were multifunctional; (ii) we provide evidence, through molecular modeling and activity tests of present-day enzymes, ancestors, and potential intermediates, that the maltase and isomaltase functions are difficult to optimize within one protein (but see also below); and (iii) we find that duplication resolved this adaptive conflict, and we find indications that positive selection might have driven key changes that optimized the minor isomaltase-like activity of the preduplication enzyme in one paralog, while the major maltase-like activity was further optimized in the other paralog.

Figure 2 and the statistical analysis in Table S3 indicate that the activity of the different enzymes changes significantly at certain points along the evolutionary path. Interestingly, the overall image that emerges suggests that the enzymes developed activity towards either maltose-like or isomaltose-like sugars, but not both. This pattern is most clear in the evolution of ancMal-Ima to ancMal and ancIma1–4. The postduplication improvement of the different activities present in the ancestral allele, with each of the new copies displaying increased activity for one type of substrate and concomitantly decreased activity towards the other substrate class, could be indicative of trade-offs in the evolution of the *MALS* gene family. However, the word “trade-off” implies that the two incompatible functions are both under selection, which is difficult to prove for the ancient enzymes. Moreover, our results indicate that for the ancient ancMalS enzyme, it is possible to simultaneously increase the activity towards both maltose-like and isomaltose-like substrates. Together, our analyses show that it is possible to optimize (to a certain extent) one function of a multifunctional enzyme without significantly reducing the other (minor) activity. However, analysis of the complete evolutionary path and molecular modeling of the active pockets of the enzymes shows that full optimization of both functions in a single enzyme is difficult to achieve, due to steric hindrance for one substrate class when fully optimizing the active pocket for binding of the other substrate type. This problem can be most easily overcome by duplication of the enzyme, allowing optimization of the different subfunctions in different paralog copies, as can be seen in the transition of ancMal-Ima to ancMal and ancIma1–4.

While most aspects of our data fit with the EAC model, some results are more difficult to reconcile with the EAC theory. Specifically, one of the pillars of the EAC model is that positive selection drives the specialization of both paralogs after duplication. While our data demonstrate that duplication of anc*MAL*-*IMA* has led to optimization of both subfunctions in different duplicate lineages (maltase-like activity in anc*MAL* and isomaltase-like activity in anc*IMA1–4*), our selection tests only reveal indications of positive selection in the anc*IMA1–4* lineage but not in the *ancMAL* lineage. Moreover, as discussed above, positive selection is difficult to prove [44],[49], and we cannot exclude the possibility of both false positive and false negative artifacts.

Recently, some other likely examples of the EAC mechanism have been described [16],[17],[50]–[52]. These studies also presented plausible arguments for ancestral multifunctionality, adaptive conflict, and/or adaptive optimization of subfunctions in different paralogs, but as in the present case, none could provide strong experimental evidence for all three predictions made by the EAC model [48],[53]. Instead of classifying the evolutionary trajectory of particular gene duplicates into one of the many models for gene duplication, it may prove more useful to distill a more general picture of duplicate evolution across a gene family that includes aspects of dosage selection, and sub- and neofunctionalization, like the one depicted in Figure 7.

Our study is the first to investigate multiple duplication events in the same gene family in detail. Interestingly, we found that evolution has taken two different molecular routes to optimize isomaltase-like activity (the evolution of anc*MAL-IMA* to anc*IMA1–4* and anc*IMA5* to *IMA5*). In both cases, only a few key mutations in the active pocket are needed to cause shifts in substrate specificity. Some of these key mutations exhibit epistatic interactions. For example, the shift in substrate specificity occurring on the path from anc*MAL*-*IMA* to anc*IMA1–4* depends in part on mutations at three co-evolving positions (218, 278, and 279), but only one mutational path (279-218-278) shows a continuous increase in isomaltase-like activity. Interestingly, there is also a different path in the opposite direction (218-279-278) that shows a continuous increase in the ancestral maltase-like activity. This implies that the complex co-evolution at these three positions may be reversible. Interestingly, a recent study of the evolutionary history of plant secondary metabolism enzymes also identified AA changes that appear to be reversible [51], in contrast to the situation for, for example, glucocorticoid receptor evolution, where evidence was found for an “epistatic ratchet” that prevents reversal to the ancestral function [54].

It is tempting to speculate that complex mechanisms like those driving the evolution of the *MALS* gene family may be a fairly common theme. Many proteins display some degree of multifunctionality or “promiscuity” [55]–[57], just like the ancestral ancMal enzyme. Moreover, directed “in vitro” protein evolution experiments have shown that novel protein functions often develop from pre-existing minor functions [58],[59]. Although the different functions within an enzyme often exhibit weak trade-offs, allowing optimization of the minor activity without affecting the original function of the enzyme [55],[59],[60], this may not always be the case. If there are stronger trade-offs between different subfunctions, duplication may enable the optimization of the conflicting functions in different paralogs.

While it is difficult to obtain accurate dating of the various duplication events, the duplication events studied here appear to postdate the divergence of *Saccharomyces* and *Kluyveromyces* clades, estimated to have occurred 150 mya [61], but predate the divergence of *Saccharomyces* and *Lachancea* and the yeast whole genome duplication, about 100 mya. *MALS* diversification may thus have happened around the appearance and spread of angiosperms (Early Cretaceous, between 140 and 100 mya [62]) and fleshy fruits (around 100 mya). Tentative dating results can be found in Table S6, but these should be approached with caution (see Text S1). The major shift in the earth's vegetation caused by the rise of the angiosperms almost certainly opened up new niches, and it is tempting to speculate that duplication and diversification of the *MALS* genes may have allowed fungi to colonize new niches containing sugars hydrolyzed by the novel Mal (Ima) alleles. In other words, the availability of novel carbon sources in angiosperms and fleshy fruits could have provided a selective pressure that promoted the retention of *MALS* duplicates and the ensuing resolution of adaptive conflicts among paralogs.

## Materials and Methods

### Phylogenetic Tree Construction

In total, the nucleotide and protein sequences of 169 extant maltases were collected for yeast species ranging from *Saccharomyces cerevisiae* to *Pichia* and *Candida* species. For *Kluyveromyces thermotolerans*, *Saccharomyces kluyveri*, and *Kluyveromyces lactis*, sequences were downloaded from Génolevures (www.genolevures.org). Sequences for many of the *Saccharomyces cerevisiae* and *Saccharomyces paradoxus* genes were obtained from the sequence assemblies provided by the Wellcome Trust Sanger Institute (http://www.sanger.ac.uk/research/projects/genomeinformatics/sgrp.html). All of the remaining extant maltase sequences were downloaded from NCBI (www.ncbi.nlm.nih.gov/). Sequences with greater than 92% pairwise protein sequence similarity to other sequences in the dataset were removed to reduce the phylogenetic complexity. All seven *Saccharomyces cerevisiae* S288c alleles were kept, however, yielding a final dataset of 50 sequences (see also Dataset S1).

We used ProtTest 2.4 [63] to score different models of protein evolution for constructing an AA-based phylogenetic tree. All possible models with all improvements implemented in the program were taken into account. An initial tree was obtained by Neighbor-Joining (BioNJ), and the branch lengths and topology were subsequently optimized for each evolutionary model independently. The LG+I+G model came out as best with a substantial lead over other protein models using −lnL, AIC, and AICc selection criteria (AICc = 43,061.26 with AICw = 1.00, while the second best model was WAG(+I+G) with AICc = 43,158.00 and AICw = 0.00). Consequently, an AA-based phylogeny for the 50 sequences was determined using MrBayes 3.1.2 [36] with a LG invariant+gamma rates model (four rate categories). Since the LG model is not implemented by default in MrBayes, we used a GTR model and fixed the substitution rate and state frequency parameters to those specified by the LG model. The BMCMC was run for 10^6^ generations, sampling every 100 generations, with two parallel runs of four chains each. A burn-in of 2,500 samples was used, and the remaining 7,501 samples were used to construct a 50% majority-rule consensus phylogeny (Figure S1). The AWTY program [64] was used to check proper MCMC convergence under the given burn-in conditions. MrBayes AA tree constructions were also performed under other evolutionary models (WAG, JTT). Additional tests were performed to exclude Long Branch Attraction (LBA) artifacts (see Text S1). We also inferred a maximum likelihood (ML) tree using PhyML under the LG+I+G model with four rate categories [37]. The initial tree was again obtained by BioNJ; tree topology, branch lengths, and rate parameters were optimized in a bootstrap analysis with 1,000 replicates.

We also used MrBayes to construct a codon-based phylogeny, using a GTR codon model of evolution. The original dataset of 50 sequences contained 18 sequences for species that employ the alternative yeast nuclear genetic code (all of them outgroup species). These sequences were removed from the dataset, resulting in a reduced dataset of 32 sequences. The codon alignment was obtained by translating the AA alignment obtained earlier. BMCMC analysis and consensus phylogeny construction were performed as described above for the AA trees. We contrasted models that did and did not allow for ω rate variation (i.e., the “Equal” versus “M3” codon model in MrBayes). AWTY analysis indicated that the latter was not able to converge properly, so we used the results of the Equal model.

### Ancestral Sequence Reconstruction

The PAML package [65] was used to infer the posterior AA probability per site in the ancestors of interest under several commonly used models of protein evolution (LG, WAG, JTT), using the corresponding Bayesian consensus phylogenies. Both marginal and joint probability reconstructions were performed. The marginal reconstructions are presented in Table S1. Protein sequences resulting from marginal reconstructions under the JTT model were used to synthetize ancestral enzymes.

### Positive Selection Tests

We performed tests for positive selection on the codon-based phylogeny obtained as described above. Various branch methods and branch-site methods included in the PAML [65] and HyPhy [66] packages were used.

#### Branch tests

We first explored the change in selective forces over time using the branch models implemented in the PAML package. The fit of the free-ratio model, which assigns an independent ω value for each branch, was found to be significantly better than that of null model assigning only one ω value to the whole tree (LRT stat = 438.43; *df* = 60; *p*<0.0001). This test confirms the presence of variability in selection pressure across branches of the codon tree, but its ω estimates are not reliable because the free-ratio model suffers from overparameterization.

We therefore applied the GABranch method, available as an extension to the HyPhy package [41],[66], as described in [67]. This method uses a genetic algorithm to search through the space of possible models and divides the branches of the phylogenetic tree in subsets of branches that share the same ω estimate, reducing parametric complexity. We used the 012034 GTR nucleotide model, selected by a HyPhy model selection routine from all 203 available GTR models. We repeated the GABranch procedure on five replicates and pooled results for postprocessing, after ensuring that all replicates reached similar solutions. The postprocessing resulted in a final branch partitioning model with four ω rate categories. Since the GABranch method itself is focused on finding the best branch-clustering scheme rather than finding the best ω estimates, the estimated ω values obtained in the GABranch analysis were further optimized using a HyPhy model optimization routine that allows for nonsynonymous rate heterogeneity. The net effect was an increase of the estimated ω values for all four rate categories (see Figure 4).

#### Branch-site tests

We used the modified branch-site model A implemented in PAML, which allows ω to vary both among sites in the sequence alignment and across branches on the tree, to screen for positive selection on sites along specific branches [42]. We used the anc*IMA*1–4, anc*MAL*, and anc*IMA*5b branches separately as the foreground branch, while the rest of the phylogeny was considered as the background, and assessed deviation from the null model (no positive selection) using a Likelihood Ratio Test following a χ_1_^2^ distribution [68]. A Bonferroni correction was employed to control for multiple testing [69], and *a posteriori* BEB inference was used to identify the sites that are most likely under positive selection [70].

We also used an alternative branch-site method that was recently implemented in the HyPhy package [43]. This method similarly identifies branches that are subject to episodic diversifying selection but differs from the branch-site tests implemented in PAML in that no background and foreground branches need to be specified a priori. Instead, the method fits a sequence of increasingly more complex models to the data, including a model that permits unrestricted combinations of selective regimes across sites and branches. Subsequently, all branches with some proportion of sites with ω>1 were tested for positive selection using a series of LRTs.

### Co-Evolving Residue Detection

Co-evolving residues in the *MALS* gene family were detected using the framework described by [71]. The NCBI Blast server was used to collect *Saccharomyces cerevisiae* S288c *MAL12* maltase homologs, with an E-value <10e-70, resulting in a set of 1,211 sequences. Proteins were removed that were shorter than 400 AAs, longer than 800 AAs, and more than 95% similar to another protein in the dataset. This resulted in a dataset of 640 maltase homologs with sequence similarity >40% compared to *Saccharomyces cerevisiae* S288c *MAL12*. These sequences were aligned with MAFFT and only the most reproducible residue–residue couplings (present in at least 90% of the splits) were retained.

### Statistical Analyses

A two-way ANOVA using log-transformed k_cat_/K_m_ (to obtain values that are normally distributed) as the variable and the different enzymes and sugars as factors was performed using the aovSufficient function from the HH package in R. This analysis was followed by pairwise comparisons using the Games-Howell post hoc test (since samples had unequal variances, as demonstrated by Levene's test). Results can be found in Table S3.

### Microbial Strains, Growth Conditions, and Molecular Techniques

Ancestral maltase genes were synthesized and cloned into vectors for overexpression in *E. coli* host cells by GENEART (www.geneart.com). Sequences can be found in Table S1 and Dataset S2. The inferred protein sequences were reverse translated in order to optimize their codon usage for *E. coli*. These gene sequences were synthesized including an N-terminal 6xHis tag (ATGGGCAGCAGCCATCATCATCATCATCACAGCAGCGGCCTGGTGCCGCGCGGCAGCCAT) and 5′UTR (TCTAGAAATAATTTTGTTTAACTTTAAGAAGGAGA TATACC), cloned into in-house vectors at GENEART, and then sequenced. Subsequently, the inserts were subcloned into pET-28(a) vectors (Merck) via XbaI/XhoI sites. All of the overexpression plasmids were transformed into *E. coli* strain BL21*. All *E. coli* strains were grown under selection in standard LB media+kanamycin (Sigma Aldrich). Details on protein expression and purification can be found in Text S1.

### Enzyme Assays and Data Analysis

The activities of the purified ancestral and present-day enzymes were determined by measuring glucose release from α-glucosides (maltose, sucrose, turanose, maltotriose, maltulose, isomaltose, palatinose, and methyl-α-glucoside) using a standard glucose oxidase/peroxidase coupled reaction. All sugars were purchased in their highest available purity. More information on the purchased sugars as well as a detailed protocol can be found in Text S1.

For each protein and substrate, the reaction velocity (amount of glucose produced per time unit) was determined. Subsequently, reaction velocities normalized by enzyme concentration as a function of substrate concentration were plotted and fitted using a nonlinear least squares fitting routine (Levenberg-Marquardt algorithm) both to Michaelis-Menten-style kinetics and Hill-style kinetics:
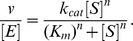
The data fits were compared using an *F* statistic (i.e., Michaelis-Menten is a specific case of Hill kinetics with *n* = 1), and the Michaelis-Menten model was rejected with α = 5%. From these fits, errors (standard deviations) were computed by jack-knifing over the individual substrate concentrations (12 data points in total). For numerical optimization, code was written in Python using NumPy. Model parameters of interest, along with their associated errors, were extracted (i.e., k_cat_ and K_m_; see Table S2). Processing (http://processing.org) was used to draw Figure 2 and Figure 5F by writing code. Enzyme efficiencies were plotted (as vertical lines) at different points on the tree, and values between were interpolated.

### Fitness Measurements

Relative Malthusian fitness was determined by competing unlabelled WT (KV1042), *mal12* (KV1151), and *mal32* (KV1153) strains against a reference strain (KV3261), expressing GFP from the *TDH3*p. Details can be found in the Supporting Information section.

### Molecular Modeling

All molecular modeling was performed using the MOE 2010.10 package (The Molecular Operating Environment, The Chemical Computing Group, Montréal, Canada). The recently released crystal structure of the Ima1 protein (pdb entry: 3A4A), with glucose in the binding pocket, was used as a template to construct the different *MALS* homology models, with implementation of the Amber99 force field. Since the AAs contacting this glucose molecule are conserved within the different *MALS* subgroups, this glucose was used to model the different sugar substrates within the active sites, using the MOE 2010.10 ligX implementation.

Full methods and any associated references can be found in the Supporting Information section.

## Supporting Information

Dataset S1 MAFFT alignment of the 50 MalS sequences (fasta format).
(ZIP)

Dataset S2 AA sequences of resurrected enzymes (fasta format).
(ZIP)

Dataset S3 MAFFT alignment of the 50 MalS sequences.
(TXT)

Dataset S4 AA sequences of resurrected enzymes.
(TXT)

Figure S1 Bayesian consensus topology of the 50 *MALS* genes. MrBayes consensus tree of the 50 *MALS* genes (AA-based, LG+I+G model with four rate categories). Posterior probabilities are indicated on the branches.
(TIF)

Figure S2 Maximum likelihood phylogeny of the 50 *MALS* genes. Maximum likelihood phylogeny of the 50 *MALS* genes calculated with PhyML (AA-based, LG+I+G model with four rate categories, 1,000 bootstraps). Bootstrap values are indicated on the branches.
(TIF)

Figure S3 Bayesian consensus topology of the 50 *MALS* genes with fast evolving sites removed. MrBayes consensus tree of the 50 *MALS* genes (AA-based, LG+I+G model with four rate categories). All AA sites with more than three variable AAs in the outgroup were stripped from the alignment. Posterior probabilities are indicated on the branches.
(TIF)

Figure S4 Bayesian consensus topology of the *MALS* genes without *K. lactis*. MrBayes consensus tree of the *MALS* genes (AA-based, LG+I+G model with four rate categories). The *K. lactis* branch was not included in the tree reconstruction. Posterior probabilities are indicated on the branches.
(TIF)

Figure S5 Bayesian consensus topology of the *MALS* genes without the outgroup. MrBayes consensus tree of the *MALS* genes (AA-based, LG+I+G model with four rate categories). The outgroup branches were not included in the tree reconstruction. Posterior probabilities are indicated on the branches.
(TIF)

Figure S6 Schematic tree showing inferred orthology–paralogy relationships between different *MALS* genes. A schematic version of the codon-based phylogenetic tree inferred with MrBayes (see Figure 4) is shown. Duplication events, D; speciation events, S. Asterisks denote nodes along a segment with ambiguous speciation/duplication history.
(TIF)

Figure S7 Structural differences between *K. lactis* [GI: 50312678] and *K. lactis* [GI:5441460] can explain lack of glucosidase activity in the latter enzyme. Cartoon representation of *K. lactis* [GI: 50312678] (A and C) and *K. lactis* [GI:5441460] (B and D) in two different orientations (A and B result in C and D, respectively, after a 90° rotation) with maltose represented as black and red spheres. Comparing the sequence of *K. lactis* [GI: 50312678] and *K. lactis* [GI:5441460] reveals the absence of five AAs in the latter protein. Mapping the position of these residues (the five AAs as well as two flanking residues are shown as yellow spheres in A and C; in B and D only the flanking residues are shown) shows that this region is located below the active site of the enzyme. Its deletion creates a larger cavity. This in turn could be compensated in the tertiary structure and explain the lack of activity detected for maltose- and isomaltose-like substrates for *K. lactis* [GI:5441460].
(TIF)

Figure S8 Crucial role for the residue at position 216 in determining substrate affinity. Structural analysis of the active site reveals a crucial role for position 216 in determining substrate affinity, by affecting the hydrophobic/hydrophilic interactions with the different substrate classes. Subpanels are graphical representations of the binding pocket, with the residue at position 216 shown as spheres. Panels A and B depict an active site with threonine at position 216, whereas C and D depict an active site with valine at position 216. Maltose (A and C) and isomaltose (B and D) are represented as sticks. This structural analysis shows that threonine is able to form a hydrogen bond with a hydroxyl of the secondary glucose in maltose (A). The secondary glucose of isomaltose, however, is positioned in such a way that it causes unfavorable interactions (B). On the other hand, when residue 216 is a valine, it can form hydrophobic interactions with isomaltose (D).The hydrophobic side chain of valine is incompatible with the hydrophilic binding mode of maltose (C).
(TIF)

Figure S9 Strains lacking one of the *MAL12/MAL32* paralogs have a fitness defect on maltose compared to wild type. *mal12* (KV1151) and *mal32* (KV1153) strains show a significant fitness defect compared to the wild-type strain (KV1042) on maltose. A *mal12 mal32* double deletion strain does not grow on maltose. Asterisks indicate significant differences between mutant and wild-type strains (α = 0.05). Error bars represent 95% confidence intervals.
(TIF)

Table S1 Results of ancestral sequence reconstruction assuming different models of protein evolution. Except for the protein evolution models used, all reconstructions were performed using the same PAML settings. The JTT reconstruction, which was used to synthesize the ancestral proteins, was performed using PAML v4.2a, while the WAG and LG reconstructions were performed using PAML v4.4c. The residue numbering in the first column follows the numbering of the *S. cerevisiae* Mal12 protein, which was used to fill gaps in the alignment that were treated as missing data by PAML. Column 2 contains the corresponding *S. cerevisiae* Ima1 residue numbering, which is used throughout the article. Subsequent columns labeled JTT, WAG, and LG contain the results of ancestral state reconstruction using the respective models of protein evolution and based on the corresponding consensus phylogenies calculated with MrBayes. For ambiguous ancestral residues (i.e., if there are multiple residues at a certain site with a posterior probability >0.20), a list with posterior probabilities is provided. If an AA has a posterior probability equal to 1 or if the ancestral AA could not be calculated due to alignment gaps (Dataset S1), no probabilities are listed.
(XLS)

Table S2 k_cat_ and K_m_ values for different enzymes on different sugars. File contains k_cat_ and K_m_ values for each enzyme for the different sugars tested. Values of K_m_ too high to be measured (due to the very low affinity of enzyme for a specific substrate) were set to 10,000. Standard deviations were computed by jack-knifing over the individual sugar concentrations. Details on measurement can be found in Text S1.
(XLS)

Table S3 Results of two-way ANOVA analysis on log-transformed k_cat_/K_m_. Activities of the different enzymes on each sugar were compared using a two-way ANOVA on log-transformed k_cat_/K_m_, followed by the Games-Howell post hoc test (taking into account the differences in variance between the different activities, as demonstrated by Levene's test).
(XLS)

Table S4 Results of PAML branch-site tests. Values in Table S4 show the result of PAML branch-site tests to identify residues that are under positive selection on three specific branches of the *MALS* phylogeny. Branch identifiers follow the nomenclature of Figure 4.
(DOC)

Table S5 Genotypes of yeast strains used in this study.
(DOC)

Table S6 Dating results for key splits in the *MALS* gene tree. Mean, median, and geometric mean refer to different average age estimates obtained from the sampled traces across the different MCMC chains, and 95% HDP upper and lower can be regarded as 95% confidence intervals (see BEAST documentation). The effective sample size (ESS) is a measure of convergence (higher is better).
(DOC)

Text S1 Full Materials and Methods.
(DOC)

## Abbreviations

AA: amino acid
AIC: Akaike information criterion
anc: ancestral
BEB: Bayes empirical Bayes
BMCMC: Bayesian Markov chain Monte Carlo
df: degree of freedom
EAC: escape from adaptive conflict
GTR: generalized time reversible
IAD: innovation-amplification-divergence
JTT: Jones, Taylor and Thornton
LBA: long branch attraction
LG+I+G: Le and Gascuel+invariable sites+gamma distributed rate heterogeneity
LRT: likelihood ratio test
MalS: maltase
ML: maximum likelihood
Ima: isomaltase
WAG: Whelan and Goldman
