# Supplementary figures and images for: Reconstruction of Ancestral Metabolic Enzymes Reveals Molecular Mechanisms Underlying Evolutionary Innovation through Gene Duplication
Source: PLoS Biol. 2012 Dec 11;10(12):e1001446. doi: 10.1371/journal.pbio.1001446 (PMC3519909; doi:10.1371/journal.pbio.1001446)

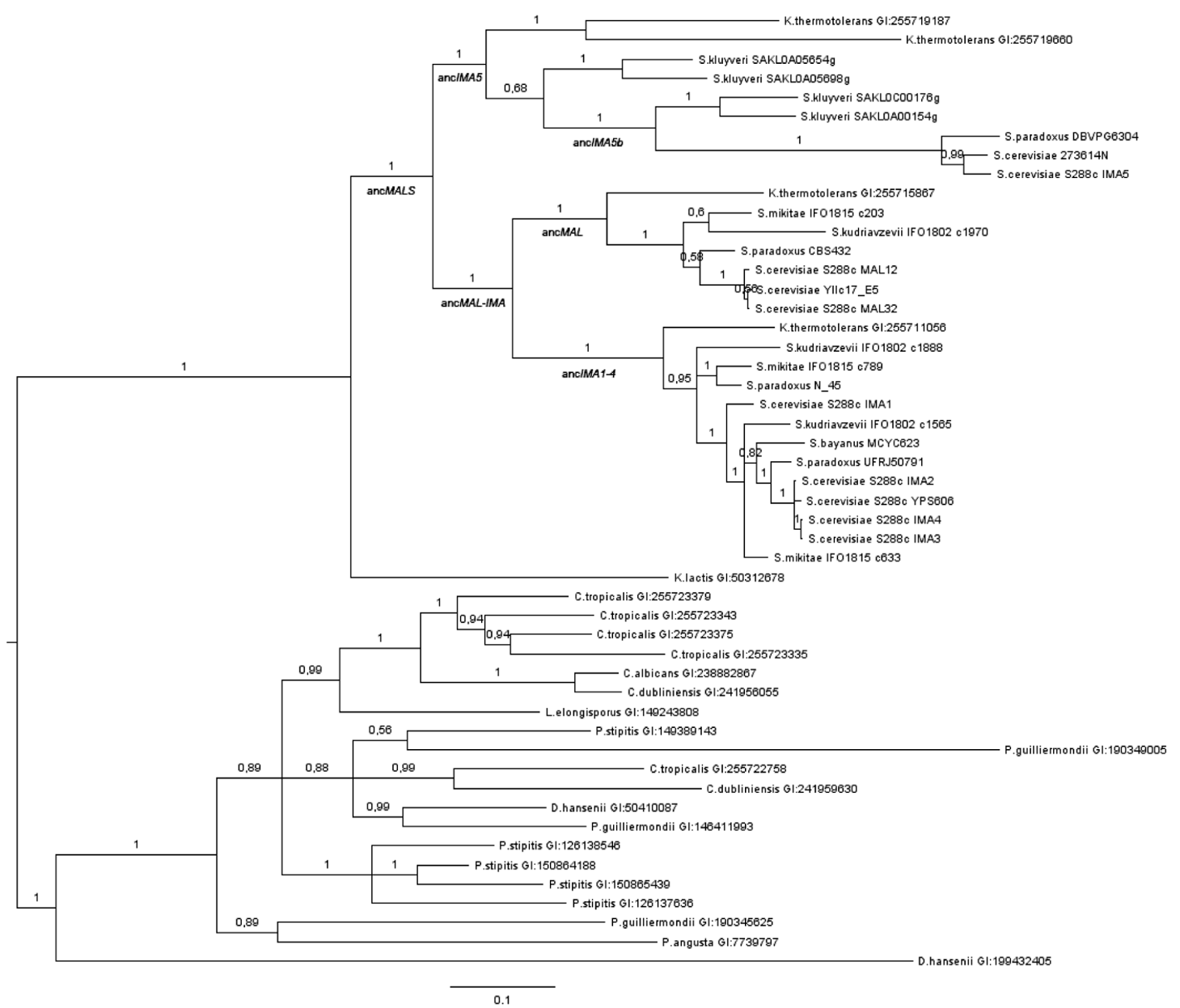

Supplement: Figure S1 — Bayesian consensus topology of the 50 MALS genes. MrBayes consensus tree of the 50 MALS genes (AA-based, LG+I+G model with four rate categories). Posterior probabilities are indicated on the branches. (TIF) [file pbio.1001446.s005.tif]

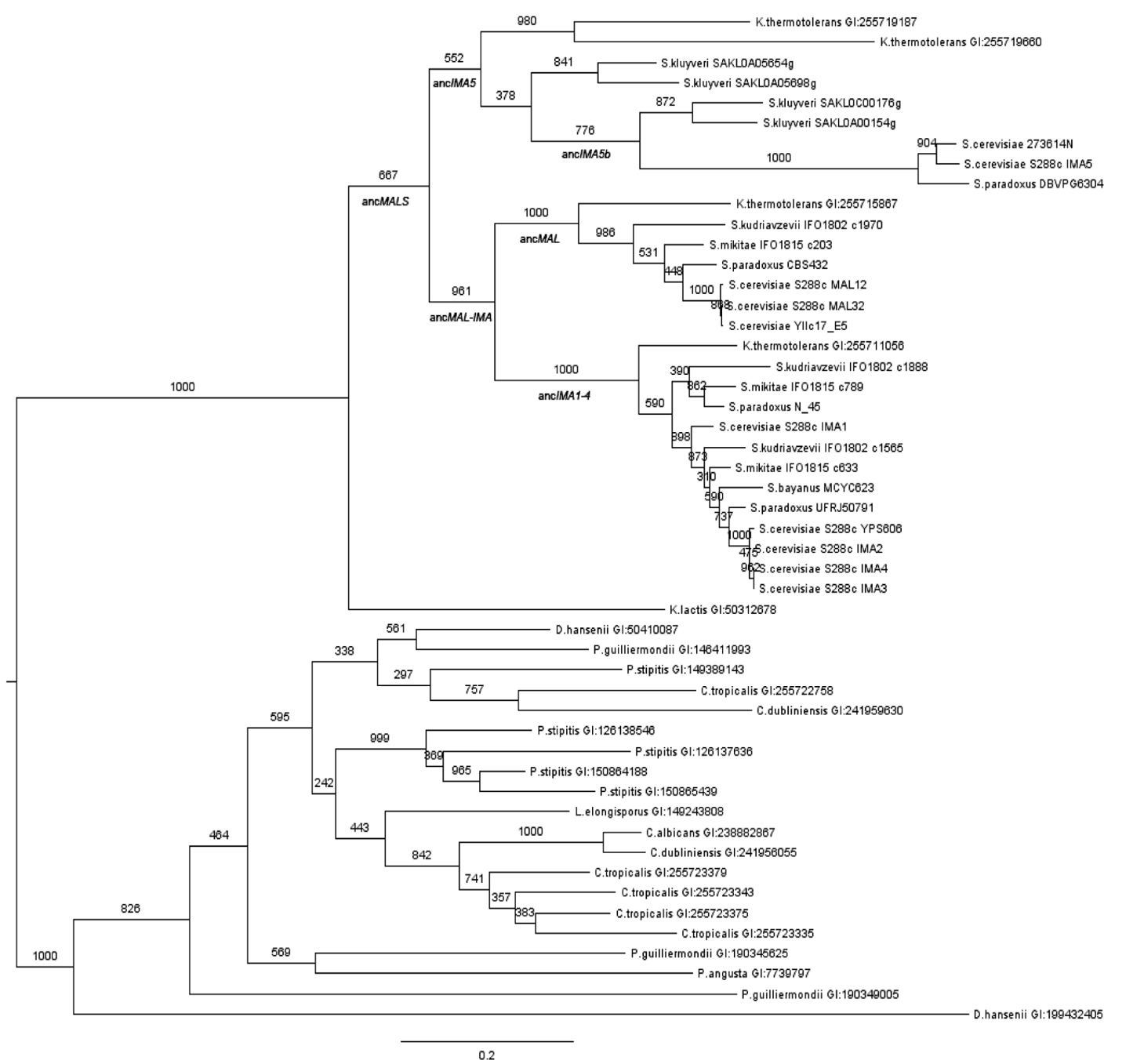

Supplement: Figure S2 — Maximum likelihood phylogeny of the 50 MALS genes. Maximum likelihood phylogeny of the 50 MALS genes calculated with PhyML (AA-based, LG+I+G model with four rate categories, 1,000 bootstraps). Bootstrap values are indicated on the branches. (TIF) [file pbio.1001446.s006.tif]

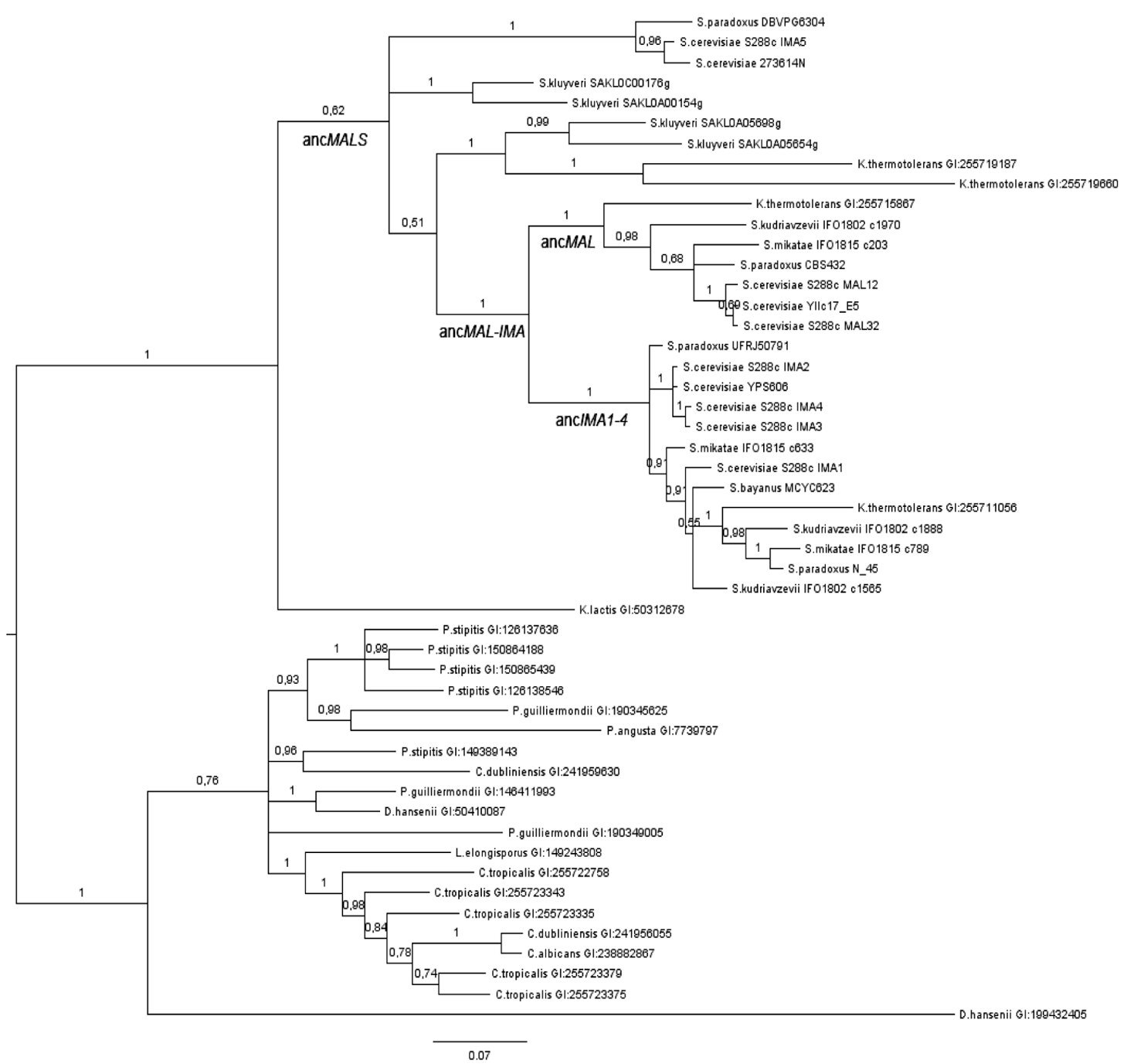

Supplement: Figure S3 — Bayesian consensus topology of the 50 MALS genes with fast evolving sites removed. MrBayes consensus tree of the 50 MALS genes (AA-based, LG+I+G model with four rate categories). All AA sites with more than three variable AAs in the outgroup were stripped from the alignment. Posterior probabilities are indicated on the branches. (TIF) [file pbio.1001446.s007.tif]

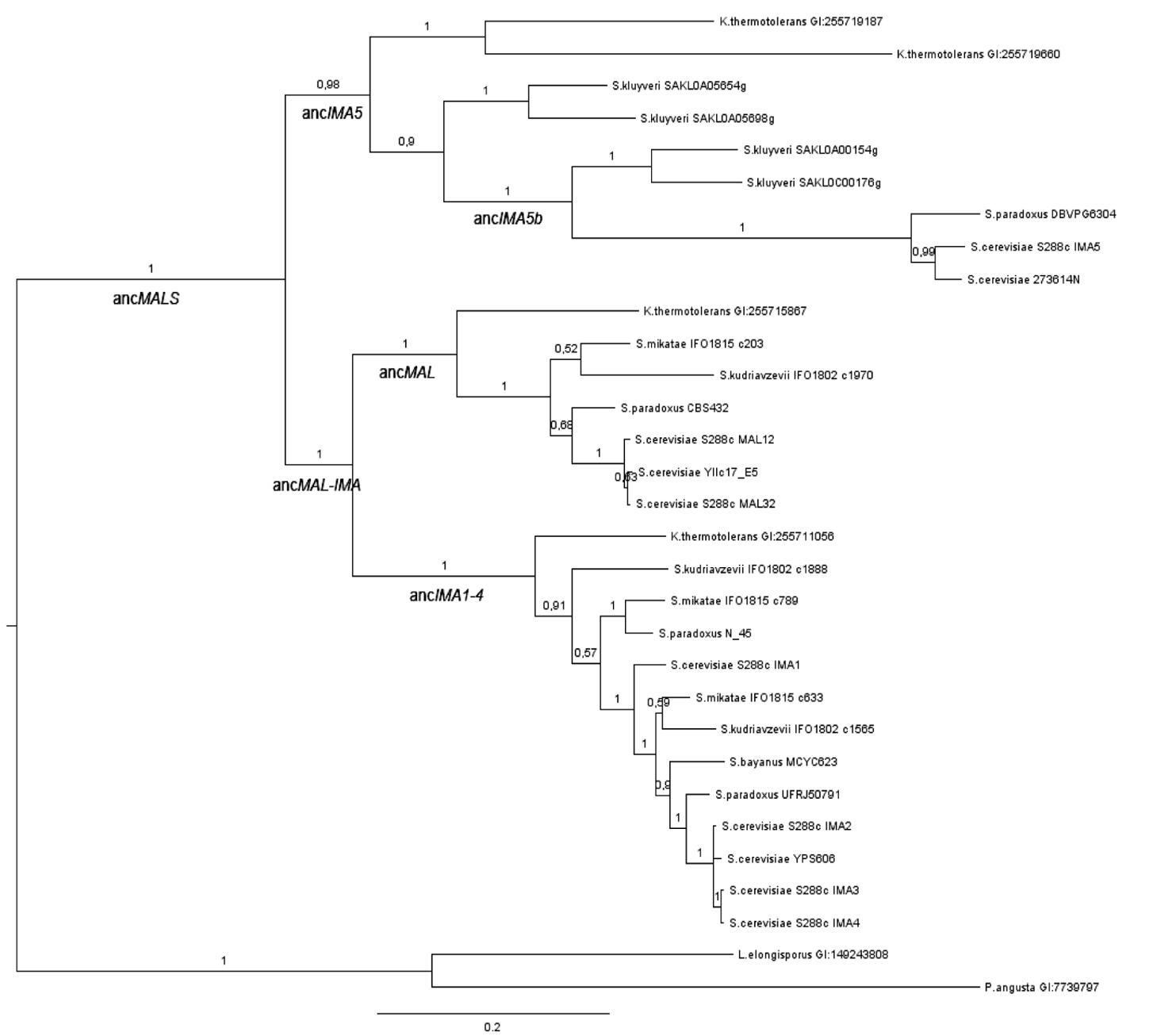

Supplement: Figure S4 — Bayesian consensus topology of the MALS genes without K. lactis. MrBayes consensus tree of the MALS genes (AA-based, LG+I+G model with four rate categories). The K. lactis branch was not included in the tree reconstruction. Posterior probabilities are indicated on the branches. (TIF) [file pbio.1001446.s008.tif]

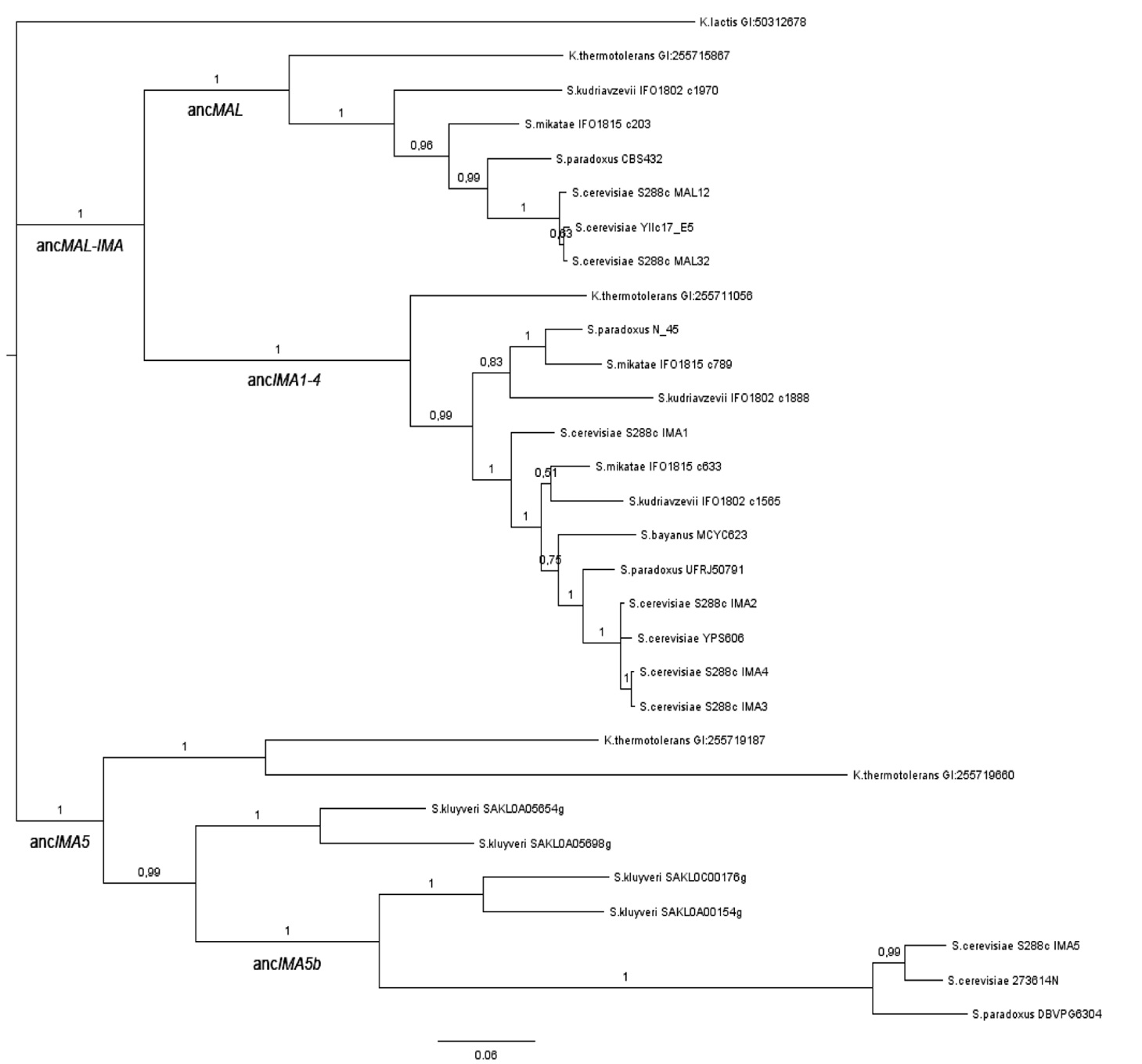

Supplement: Figure S5 — Bayesian consensus topology of the MALS genes without the outgroup. MrBayes consensus tree of the MALS genes (AA-based, LG+I+G model with four rate categories). The outgroup branches were not included in the tree reconstruction. Posterior probabilities are indicated on the branches. (TIF) [file pbio.1001446.s009.tif]

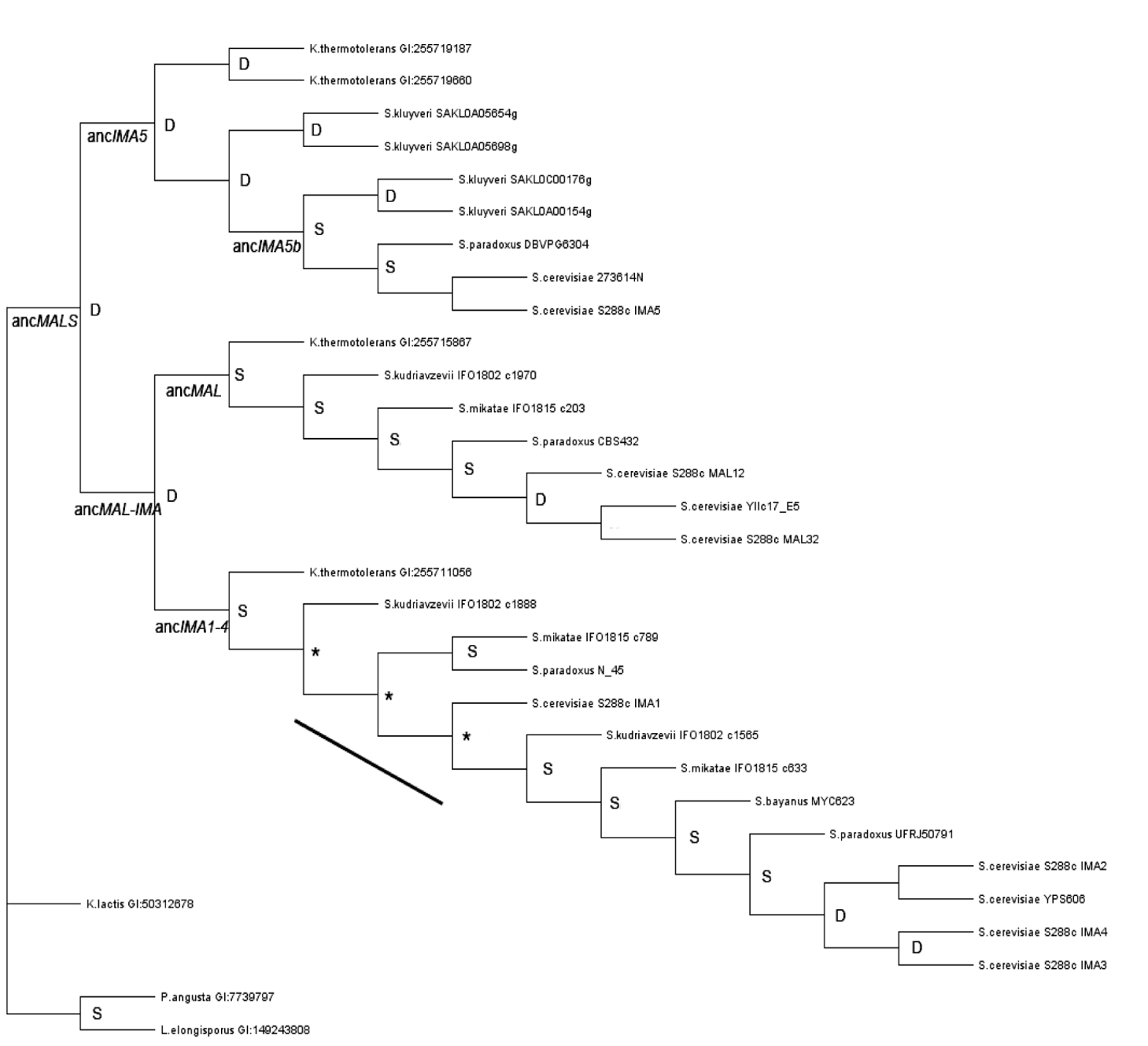

Supplement: Figure S6 — Schematic tree showing inferred orthology–paralogy relationships between different MALS genes. A schematic version of the codon-based phylogenetic tree inferred with MrBayes (see Figure 4) is shown. Duplication events, D; speciation events, S. Asterisks denote nodes along a segment with ambiguous speciation/duplication history. (TIF) [file pbio.1001446.s010.tif]

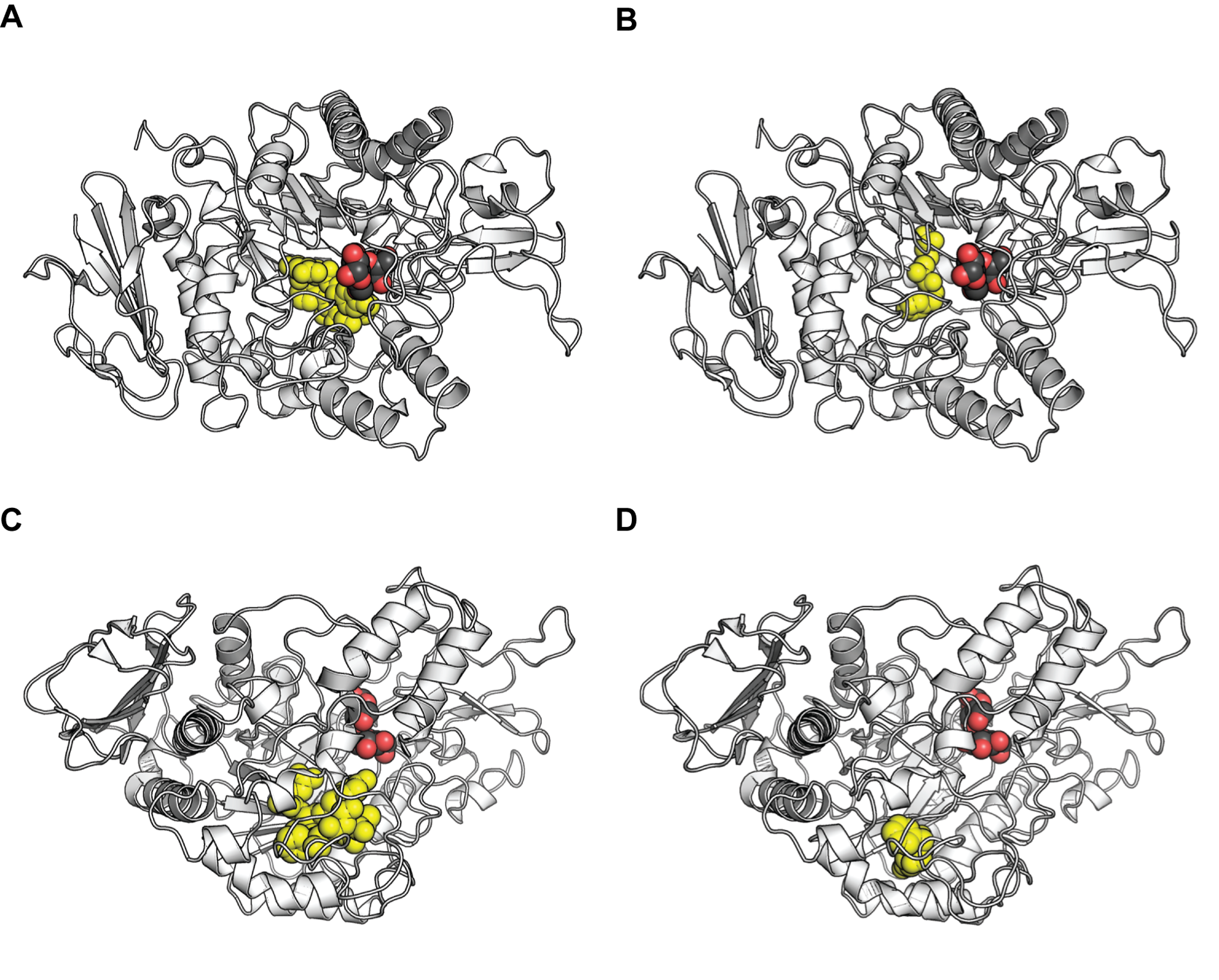

Supplement: Figure S7 — Structural differences between K. lactis [GI: 50312678] and K. lactis [GI:5441460] can explain lack of glucosidase activity in the latter enzyme. Cartoon representation of K. lactis [GI: 50312678] (A and C) and K. lactis [GI:5441460] (B and D) in two different orientations (A and B result in C and D, respectively, after a 90° rotation) with maltose represented as black and red spheres. Comparing the sequence of K. lactis [GI: 50312678] and K. lactis [GI:5441460] reveals the absence of five AAs in the latter protein. Mapping the position of these residues (the five AAs as well as two flanking residues are shown as yellow spheres in A and C; in B and D only the flanking residues are shown) shows that this region is located below the active site of the enzyme. Its deletion creates a larger cavity. This in turn could be compensated in the tertiary structure and explain the lack of activity detected for maltose- and isomaltose-like substrates for K. lactis [GI:5441460]. (TIF) [file pbio.1001446.s011.tif]

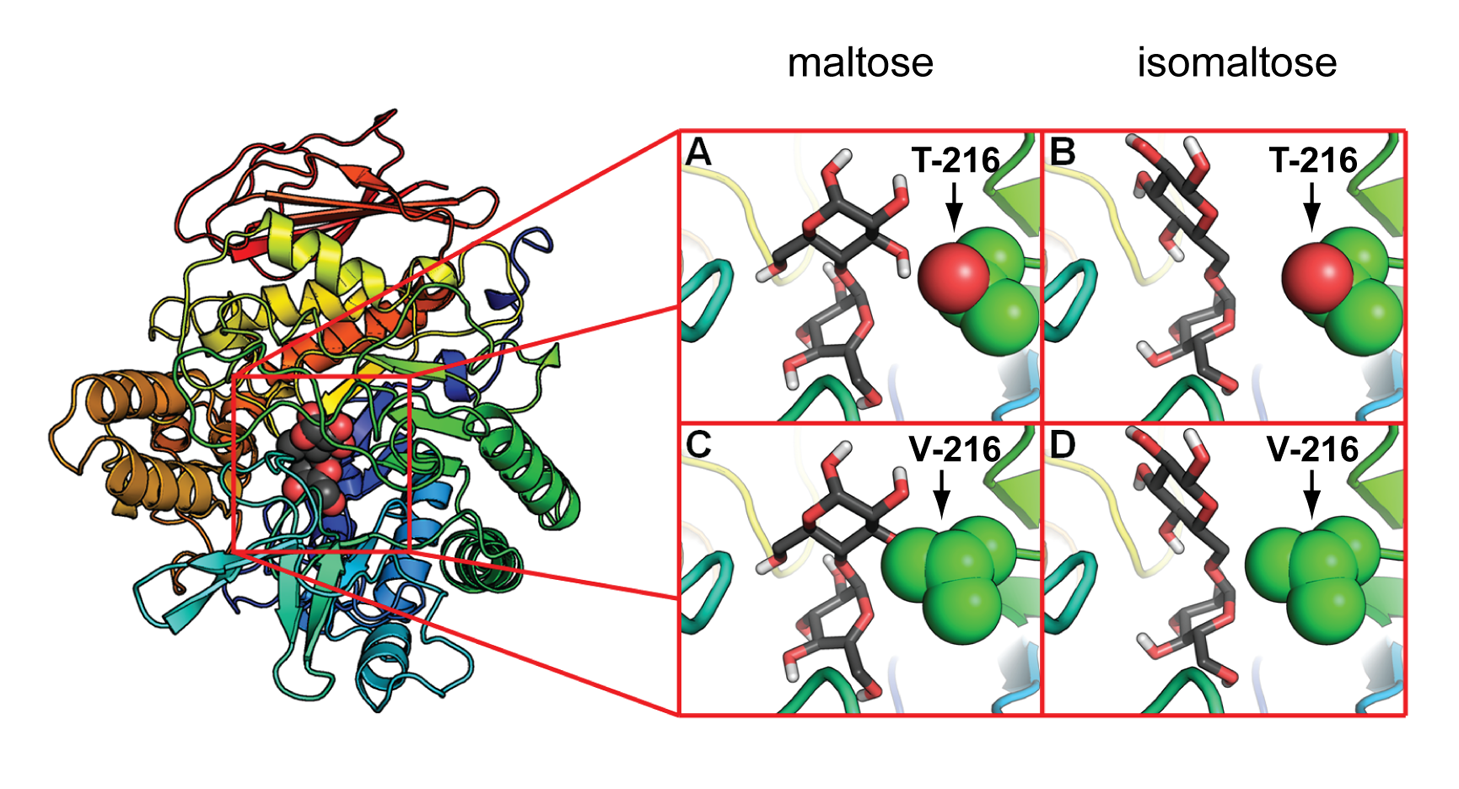

Supplement: Figure S8 — Crucial role for the residue at position 216 in determining substrate affinity. Structural analysis of the active site reveals a crucial role for position 216 in determining substrate affinity, by affecting the hydrophobic/hydrophilic interactions with the different substrate classes. Subpanels are graphical representations of the binding pocket, with the residue at position 216 shown as spheres. Panels A and B depict an active site with threonine at position 216, whereas C and D depict an active site with valine at position 216. Maltose (A and C) and isomaltose (B and D) are represented as sticks. This structural analysis shows that threonine is able to form a hydrogen bond with a hydroxyl of the secondary glucose in maltose (A). The secondary glucose of isomaltose, however, is positioned in such a way that it causes unfavorable interactions (B). On the other hand, when residue 216 is a valine, it can form hydrophobic interactions with isomaltose (D).The hydrophobic side chain of valine is incompatible with the hydrophilic binding mode of maltose (C). (TIF) [file pbio.1001446.s012.tif]

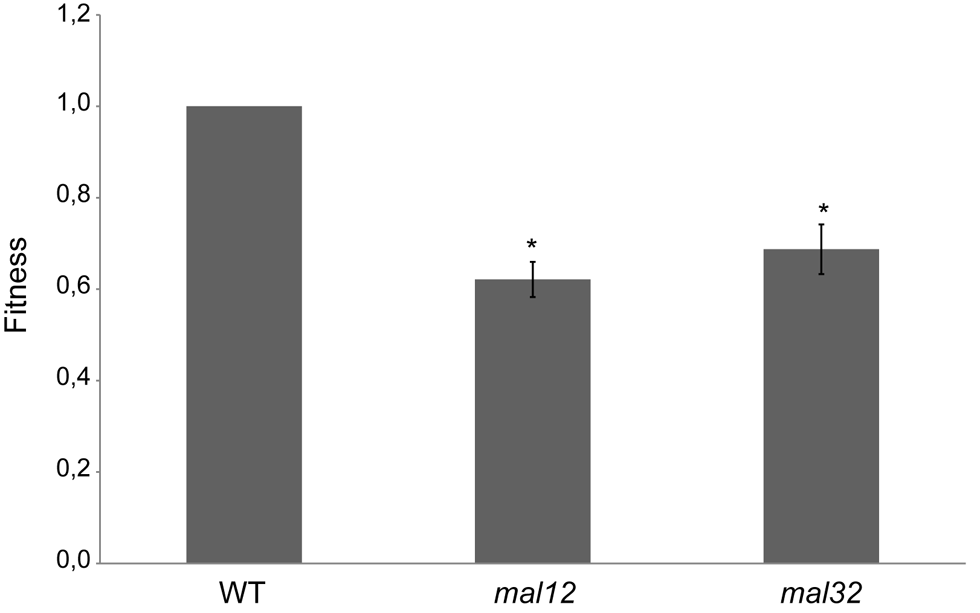

Supplement: Figure S9 — Strains lacking one of the MAL12/MAL32 paralogs have a fitness defect on maltose compared to wild type. mal12 (KV1151) and mal32 (KV1153) strains show a significant fitness defect compared to the wild-type strain (KV1042) on maltose. A mal12 mal32 double deletion strain does not grow on maltose. Asterisks indicate significant differences between mutant and wild-type strains (α = 0.05). Error bars represent 95% confidence intervals. (TIF) [file pbio.1001446.s013.tif]
