# Supplementary material for: Reconstruction of Ancestral Metabolic Enzymes Reveals Molecular Mechanisms Underlying Evolutionary Innovation through Gene Duplication
Source: PLoS Biol. 2012 Dec 11;10(12):e1001446. doi: 10.1371/journal.pbio.1001446 (PMC3519909; doi:10.1371/journal.pbio.1001446)
Supplement: Table S4 — Results of PAML branch-site tests. Values in Table S4 show the result of PAML branch-site tests to identify residues that are under positive selection on three specific branches of the MALS phylogeny. Branch identifiers follow the nomenclature of Figure 4. (DOC) [file pbio.1001446.s017.doc]

***Table S4*: Results of PAML branch-site tests, related to Figure 4.**

Values in Table S4 show the result of PAML branch-site tests to identify residues that are under positive selection on three specific branches of the *MALS* phylogeny. Branch identifiers follow the nomenclature of Figure 4.

| branch | H_0_ | H_A_ | LRT | p-value | parameter estimates | pos. selected sites* (BEB > 0.95) |
| --- | --- | --- | --- | --- | --- | --- |
| anc*IMA1-4* | -28326.54 | -28320.88 | 11.32 | p < 0.01 | 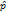_0_ = 0.934, 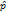_1_= 0.028,  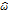_0_ = 0.082, 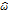_2_ = 5.466 | 216, 279, 333, 562 |
| anc*MAL* | -28334.80 | -28333.21 | 3.18 | p = 0.22 | 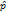_0_ = 0.953, 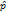_1_ = 0.029,  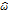_0_ = 0.083, 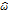_2_  = 4.738 | n/a |
| anc*IMA5*b | -28330.08 | -28322.96 | 14.24 | p < 0.001 | 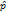_0_ = 0.950, 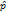_1_ = 0.029,  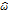_0_ = 0.083, 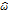_2_  = 11.245 | 216, 299, 315, 414 |
| *Based on Ima1 numbering | | | | | | |
