# Supplementary material for: Reconstruction of Ancestral Metabolic Enzymes Reveals Molecular Mechanisms Underlying Evolutionary Innovation through Gene Duplication
Source: PLoS Biol. 2012 Dec 11;10(12):e1001446. doi: 10.1371/journal.pbio.1001446 (PMC3519909; doi:10.1371/journal.pbio.1001446)
Supplement: Table S5 — Genotypes of yeast strains used in this study. (DOC) [file pbio.1001446.s018.doc]

***Table S5*: Genotypes of yeast strains used in this study.**

| **Strain name** | **Genotype** | **Ref.** |  |
| --- | --- | --- | --- |
| KV1042 | S288c Mata MAL13::HYG-RM11_MAL63c9 | a |  |
| KV1444 | S288c Mata MAL13::HYG-RM11_MAL63c9 TEFp-IMA5 | b |  |
| KV2498 | S288c Mata MAL13::HYG-RM11_MAL63c9 IMA5::KanMX | a |  |
| KV1151 | S288c Mata MAL13::HYG-RM11_MAL63c9 MAL12::KanMX | a |  |
| KV1153 | S288c Mata MAL13::HYG-RM11_MAL63c9 MAL32::KanMX | a |  |
| KV1774 | S288c Mata MAL13::HYG-RM11_MAL63c9 MAL12::KanMx MAL32::KanMX | a |  |
| KV3261 | S288c Mata MAL13::HYG-RM11_MAL63c9 TDH3p::GFP-KanMX | c |  |
| KV3002 | *Lodderomyces elongisporus* CBS2605 |  |  |
| KV1983 | *Ashbya gossypii* ATCC 10895 |  |  |
| KV3000 | *Kluyveromyces lactis* ATCC 8585 |  |  |
| KV3190 | *Saccharomyces kluyveri* CBS3082 |  |  |
| KV3191 | *Lachancea waltii* CBS6430 |  |  |
| KV2817 | *Kluyveromyces thermotolerans* CHCC5657 |  |  |
| KV3192 | *Kluyveromyces polysporus* CBS263 |  |  |
| KV3193 | *Saccharomyces castellii* CBS4309 |  |  |
| KV1980 | *Candida glabrata* CBS138 |  |  |
| KV1556 | *Saccharomyces bayanus* CBS7001 |  |  |
| KV1981 | *Saccharomyces kudriavzevii* IFO 1802 |  |  |
| KV1982 | *Saccharomyces mikatae* IFO 1815 |  |  |
| KV1557 | *Saccharomyces paradoxus* NCYC2600 |  |  |

a) Brown CA, Murray AW, Verstrepen KJ (2010) Rapid expansion and functional divergence of subtelomeric gene families in yeasts. Curr Biol 20: 895-903.

b) Overexpression construct was created using plasmid pYM-N18, containing the TEF promoter

c) Fluorescent marker was introduced in an intergenic region of chromosome II (see Smukalla S, Caldara M, Pochet N, Beauvais A, Guadagnini S, et al. (2008) FLO1 is a variable green beard gene that drives biofilm-like cooperation in budding yeast. Cell 135: 726-737.)
