# Supplementary material for: Reconstruction of Ancestral Metabolic Enzymes Reveals Molecular Mechanisms Underlying Evolutionary Innovation through Gene Duplication
Source: PLoS Biol. 2012 Dec 11;10(12):e1001446. doi: 10.1371/journal.pbio.1001446 (PMC3519909; doi:10.1371/journal.pbio.1001446)
Supplement: Table S6 — Dating results for key splits in the MALS gene tree. Mean, median, and geometric mean refer to different average age estimates obtained from the sampled traces across the different MCMC chains, and 95% HDP upper and lower can be regarded as 95% confidence intervals (see BEAST documentation). The effective sample size (ESS) is a measure of convergence (higher is better). (DOC) [file pbio.1001446.s019.doc]

***Table S6*: Dating results for key splits in the *MALS* gene tree, related to Figure 2, Figure 4 and Figure 7**

Mean, median and geometric mean refer to different average age estimates obtained from the sampled traces across the different MCMC chains, and 95% HDP upper and lower can be regarded as 95% confidence intervals (see BEAST documentation). The effective sample size (ESS) is a measure of convergence (higher is better).

|  |  |  |  |  |  |  |  |
| --- | --- | --- | --- | --- | --- | --- | --- |
|  |  |  |  |  |  |  |  |
|  |  |  |  |  |  |  |  |
|  |  |  |  |  |  |  |  |
|  | ancIMA1-4 | ancIMA5 | ancMALS | ancMAL-IMA | calibration2 | calibration1 | ancMAL |
| mean | 55,9373 | 94,1671 | 118,6754 | 87,9487 | 170,155 | 149,5962 | 55,5027 |
| stderr of mean | 9,1298E-2 | 9,8827E-2 | 8,6328E-2 | 0,102 | 1,5688E-2 | 1,5756E-2 | 0,1055 |
| median | 55,3395 | 94,348 | 119,4 | 87,8065 | 170,1588 | 149,5978 | 54,8716 |
| geometric mean | 55,3002 | 93,5564 | 118,2222 | 87,2616 | 170,1289 | 149,5666 | 54,603 |
| 95% HPD lower | 39,5439 | 73,1691 | 97,9651 | 66,8271 | 164,3415 | 143,7635 | 36,9593 |
| 95% HPD upper | 72,4399 | 114,4806 | 137,1406 | 109,0841 | 176,0083 | 155,4541 | 75,6895 |
| auto-correlation time (ACT) | 41719,8218 | 31274,6714 | 25781,4103 | 31386,5077 | 10000 | 10115,93 | 40035,6677 |
| effective sample size (ESS) | 8629,9506 | 11512,1913 | 13965,101 | 11471,1711 | 36004 | 35591,3891 | 8992,981 |
