# Supplementary material for: Reconstruction of Ancestral Metabolic Enzymes Reveals Molecular Mechanisms Underlying Evolutionary Innovation through Gene Duplication
Source: PLoS Biol. 2012 Dec 11;10(12):e1001446. doi: 10.1371/journal.pbio.1001446 (PMC3519909; doi:10.1371/journal.pbio.1001446)
Supplement: Text S1 — Full Materials and Methods. (DOC) [file pbio.1001446.s020.doc]

**Text S1: Full Materials and Methods**

**Additional tests to exclude Long Branch Attraction (LBA) artifacts**

Despite high support for our inferred topology by both Bayesian and Maximum Likelihood methods; the position of *K. lactis GI: 50312678* warranted further investigation. Our topology of the *MALS* gene family supports that this gene branched off before the *S. kluyveri* - *S. cerevisiae* split. However, according to another commonly accepted view of ascomycete evolution, the *Kluyveromyces* (*K. lactis*) and *Lachancea* (*S. kluyveri* and *K. thermotolerans*) clades branched off together from *Saccharomyces* [1]. The topology of the ascomycete species tree is currently insufficiently resolved to be considered final, and earlier studies have provided conflicting results regarding the branching order of the *Saccharomyces*, *Lachancea* and *Kluyveromyces* clades (e.g.[1,2,3,4])*.* Nevertheless, the position of the *K. lactis* branch in our topology could potentially have been impacted by long branch attraction (LBA) between the *K. lactis* branch and long outgroup branches [5]. Since *K. lactis* serves as most recent outgroup to the anc*MALS* clade, it has a big influence on ancestral sequence reconstruction and requires confidence in its placement. Bayesian and maximum likelihood methods as used in our tree reconstruction have been found to be less susceptible to LBA artifacts but nevertheless are not invulnerable to it. Improved taxon sampling around the *K. lactis* branch could mitigate possible LBA artifacts [5], but this proved impossible as all relevant *MALS* sequences known to date were already included in the tree reconstruction. We therefore ran 2 extra analyses that help to detect LBA artifacts. First, we removed all fast evolving sites in our protein alignment by discarding all sites in the alignment that had more than 3 variable amino acids in the outgroups (defined here as all sequences not belonging to *Saccharomyces/Lachancea/Kluyveromyces* species). A different placement of the *K. lactis* branch would then be indicative of LBA artifacts caused by fast evolving sites in the alignment [5]. The phylogeny was determined using MrBayes 3.1.2 with a LG+I+G model with 4 rate categories, as described above. The resulting topology (Figure S3) is consistent with the topology presented in Figures S1 and S2. Although confidence in more recent splits is lower and results in more multifurcations (most likely due to the loss of information associated with removing data from the alignment), *K. lactis* still branches off before the *S. kluyveri* - *S. cerevisiae* split with high posterior probabililty. For the second analysis, we ran 2 separate phylogenies with *K. lactis* and outgroup sequences excluded, respectively. Excluding one of the 2 potential long branch attractors should result in a correct placement of the other branch and is therefore also indicative of LBA artifacts [5]. Both phylogenies were constructed using MrBayes 3.1.2 as described in the Materials and Methods section of the main text. Figure S4 presents the phylogeny with the *K. lactis* branch removed and outgroup representatives included. The topology of the ingroup corresponds with the topology of the ingroup in figures S1 and S2 for all major splits. Figure S5 presents the phylogeny with the *K. lactis* branch included and the outgroups removed. The topology of the ingroup again corresponds almost completely with those presented in figures S1 and S2. The *K. lactis* branch multifurcates together with the anc*IMA5* and anc*MAL-IMA* clades but is not pulled inside one of the *Lachancea* clades. In conclusion, both excluding fast evolving sites and excluding potential long branch attractors did not change the major ingroup topology of the *MALS* genes (i.e. the anc*MALS* clade in figure 4) and provide support that *K. lactis GI: 50312678* indeed does not belong to one of the three *Lachancea* - *Saccharomyces* clades in the *MALS* gene phylogeny.

**Dating of *MALS* duplications**

We estimated the age of the major divergences in the *MALS* phylogeny (i.e. anc*MALS*, anc*IMA5*, anc*MAL*, anc*MAL-IMA*, and anc*IMA1-4*) using a Bayesian approach as implemented in the BEAST v1.6.1 program [6]. We employed the general GTR+I+G model of DNA substitution with four rate categories. For the clock model, we selected the lognormal relaxed-clock model, which allows rates to vary among branches without any *a priori* assumption of autocorrelation between adjacent branches. For the tree prior, we employed a Yule process of speciation, with the topology specified as in Figure 4 of the main text (without branch lengths specified). The ingroup was considered monophyletic with respect to the outgroup consisting of *P. angusta* and *L. elongisporus.* The posterior distribution of the estimated divergence times was obtained by specifying 2 calibration points based on literature. The first calibration point is the divergence of the *Saccharomyces* from the *Kluyveromyces* clade, estimated at 150 mya [7]. The second calibration point is the divergence of *C. albicans* from *S. cerevisiae*, estimated at 170 mya [8]. Both of these calibration points are however molecular-based age estimates themselves, instead of fossil and/or geological-derived. They are therefore prone to biases induced by the possible inadequacy of the molecular data, the model of molecular evolution, and the methods used to derive these estimates. Use of such calibration points in divergence dating is therefore generally discouraged but nonetheless required in this case since no other viable calibration points were available [9]. Results should however be interpreted with due caution in the present context. For both calibration age estimates, we used a normal prior with as mean the estimate and as standard deviation 3 mya. In total, 4 independent MCMC runs were run for 100 million generations, sampling every 10000 generations to reach a total of 10000 samples per individual run. Log files from each run were analyzed with Tracer v1.5 [10] using a burn-in of one million generations, and demonstrated strong equilibrium with effective sample sizes (ESS) of all parameters far exceeding 200. Convergence of run replicates was confirmed by visual inspection of traces within and between traces, and the results of the combined traces are presented in Table S6.

**Microbial strains, growth conditions and molecular techniques**

**Protein expression**

Overnight cultures of *E. coli* were diluted 1:20 into 500 mL of LB + kanamycin. These cultures were grown at 37°C for 3 hours, after which cells were induced with 1mM IPTG (Sigma Aldrich) and then grown at 30°C for another 5 hours. Cells were harvested by spinning at 6000g for 10 minutes at 4°C. The cell pellets were then frozen at -80°C.

**Protein purification**

Frozen cell pellets were thawed and resuspended in 10 mL of Eq. Wash Buffer (50 mM phosphate + 300 mM NaCl + 5% glycerol at pH 7). Cell suspensions were incubated with gentle agitation at room temperature with 7.5 mg lysozyme (Sigma Aldrich) for 15 minutes. The cell suspensions were sonicated 4x1 minute with 1 minute breaks on ice in between. The raw cell lysate was fractioned into 2 mL test tubes and spun at 10,000g for 10 minutes at 4°C. The supernatant was added to 6 mL of pre-equilibrated (3 mL packed bead volume washed twice with 15 mL of Eq. Wash Buffer) TALON (Westburg) resin in a 5 mL polypropylene column (Qiagen). The column was incubated at room temperature with gentle agitation for 20 minutes in order to bind the 6xHis-tagged proteins. After binding the resin, the column was washed twice by incubating at room temperature with gentle agitation for 10 minutes with 15 mL of Eq. Wash Buffer. The bound protein was eluted with 2.5 mL of Elution Buffer (Eq. Wash Buffer + 200 mM imidazole (Sigma Aldrich)) by incubating for 10 minutes at room temperature. The protein concentration was quantified by using a Protein Quantification Kit-Rapid (Fluka) and qualified by running on a NuPage Novex Bis-Tris Mini Gel (Invitrogen).

**Enzyme assays and data analysis**

The following sugars were purchased from Sigma in their highest available purity (number in brackets corresponds to catalogue number): maltose (M5885), sucrose (84097), turanose (T2754), maltotriose (M8378), maltulose (50796), melezitose (M5375), methyl-α-glucoside (M9376), isomaltose (I7253) and palatinose (P2007).

For maltose, sucrose, turanose, maltotriose, and maltulose, stock concentrations of 0.5, 1, 2, 3, 5, 10, 15, 20, 40, 60, 80, and 100 mM were prepared in Enzyme Assay Buffer (50 mM phosphate buffer + 300 mM NaCl + 5% glycerol at pH 6). For methyl-α-glucoside, isomaltose and palatinose, stock concentrations of 5, 10, 20, 30, 40, 50, 75, 100, 125, 150, 175, and 200 mM were prepared in Enzyme Assay Buffer. Reaction mixtures were prepared by adding 3 μL of purified protein to 27 μL of stock sugar solution in a 96-well plate, such that the final concentration of protein was ~ 100 μg/mL. The reaction plates were incubated at 30°C from 15 – 30 minutes (depending on activity of the enzyme tested), then inactivated at 98°C for 2 minutes. The final glucose concentration was measured by adding 90 μL of GOD-PAP reagent (Dialab), incubating at 30°C for 10 minutes, and measuring the absorbance at 505 nm. A negative control of *E. coli* strain BL21* (purified equivalently to the other proteins), incubated with the sugars, was included for each substrate concentration. The values obtained with this negative control were subtracted from the values obtained with the purified enzymes. The concentration of hydrolyzed substrate was determined by normalizing the measured glucose concentration by the number of glucose molecules per substrate, assuming that all glucose molecules liberated are assayable (*e.g.* for maltose divide measured concentration of glucose by 2).

**Fitness measurements**

Cultures were inoculated with equal numbers of labeled reference and unlabeled strains (~ 10^6^ cells of each) and allowed to grow for several generations. The experiment was carried out in SC maltose (2%) medium. The ratio of the two competitors was quantified at the initial and final time points by flow cytometry. Measurements were corrected for the small percentage of labeled, non-fluorescent cells that occurred even when the reference strain was cultured separately as well as for the cost of GFP expression in the labeled reference strain. This correction is made before feeding data in the S formula. For each fitness measurement, three independent replicates were performed. The selective advantage, s, of each strain was calculated as s= (ln(U_f_/R_f_)-ln (U_i_/R_i_))/T where U and R are the numbers of unlabeled and reference strain respectively, the subscripts refer to final and initial populations and T is the number of generations that reference cells have proliferated during the competition. The fitness of the unlabeled WT strain was designated 1, and the fitness of *mal12* and *mal32* strains is 1+s.

**Supplemental references**
